# Design, Synthesis, Anticancer Evaluation, Enzymatic Assays, and a Molecular Modeling Study of Novel Pyrazole–Indole Hybrids

**DOI:** 10.1021/acsomega.1c01604

**Published:** 2021-04-29

**Authors:** Ashraf S. Hassan, Gaber O. Moustafa, Hanem M. Awad, Eman S. Nossier, Mohamed F. Mady

**Affiliations:** † Organometallic and Organometalloid Chemistry Department, National Research Centre, Dokki, Cairo 12622, Egypt; ‡ Peptide Chemistry Department, National Research Centre, Dokki, Cairo 12622, Egypt; § Department of Tanning Materials and Leather Technology, National Research Centre, Dokki, Cairo 12622, Egypt; ∥ Department of Pharmaceutical Chemistry, Faculty of Pharmacy (Girls), Al-Azhar University, Cairo 11754, Egypt; ⊥ Department of Chemistry, Bioscience and Environmental Engineering, Faculty of Science and Technology, University of Stavanger, N-4036 Stavanger, Norway; # Green Chemistry Department, National Research Centre, Dokki, Cairo 12622, Egypt

## Abstract

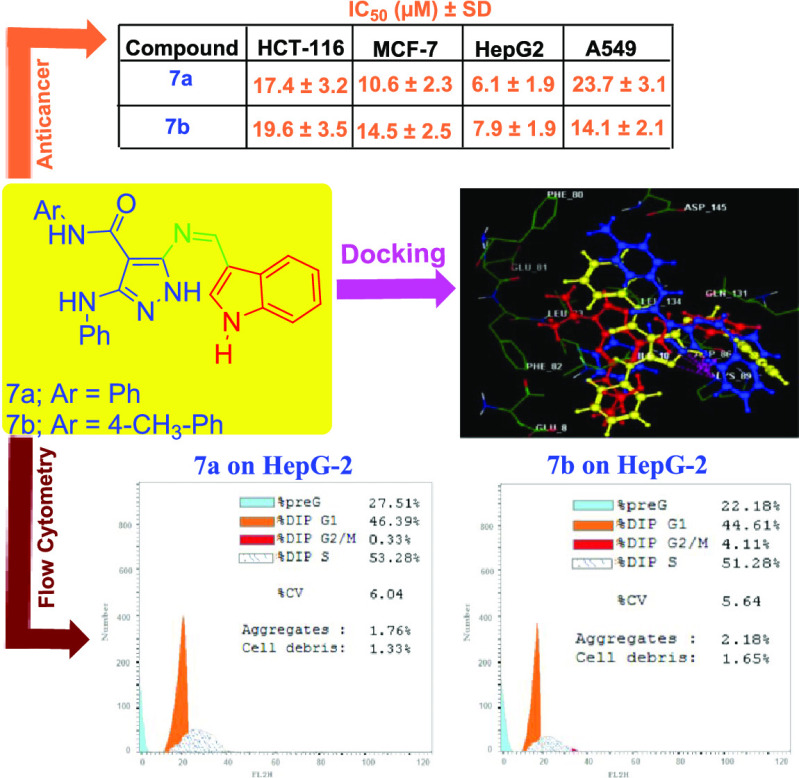

The molecular hybridization concept has recently emerged as a powerful approach in drug discovery. A series of novel indole derivatives linked to the pyrazole moiety were designed and developed *via* a molecular hybridization protocol as antitumor agents. The target compounds (**5a**–**j** and **7a**–**e**) were prepared by the reaction of 5-aminopyrazoles (**1a**–**e**) with *N*-substituted isatin (**4a**,**b**) and 1*H*-indole-3-carbaldehyde (**6**), respectively. All products were characterized *via* several analytical and spectroscopic techniques. Compounds (**5a**–**j** and **7a**–**e**) were screened for their cytotoxicity activities *in vitro* against four human cancer types [human colorectal carcinoma (HCT-116), human breast adenocarcinoma (MCF-7), human liver carcinoma (HepG2), and human lung carcinoma (A549)] using the MTT assay. The obtained results showed that the newly synthesized compounds displayed good-to-excellent antitumor activity. For example, 5-((1*H*-indol-3-yl)methyleneamino)-*N*-phenyl-3-(phenylamino)-1*H*-pyrazole-4-carboxamide (**7a**) and 5-((1*H*-indol-3-yl)methyleneamino)-3-(phenylamino)-*N*-(4-methylphenyl)-1*H*-pyrazole-4-carboxamide (**7b**) provided excellent anticancer inhibition performance against the HepG2 cancer cell line with IC_50_ values of 6.1 ± 1.9 and 7.9 ± 1.9 μM, respectively, compared to the standard reference drug, doxorubicin (IC_50_ = 24.7 ± 3.2 μM). The two powerful anticancer compounds (**7a** and **7b**) were further subjected to cell cycle analysis and apoptosis investigation in HepG2 using flow cytometry. We have also studied the enzymatic assay of these two compounds against some enzymes, namely, caspase-3, Bcl-2, Bax, and CDK-2. Interestingly, the molecular docking study revealed that compounds **7a** and **7b** could well embed in the active pocket of the CDK-2 enzyme *via* different interactions. Overall, the prepared pyrazole–indole hybrids (**7a** and **7b**) can be proposed as strong anticancer candidate drugs against various cancer cell lines.

## 1 Introduction

Cancer is one of the significant health problems and the second reason for deaths globally. Liver, breast, and lung are among the most common types of cancer diseases. Several ways have been discovered and reported for inhibiting cancer diseases, such as surgery, chemotherapy, radiation therapy, targeted therapy, immunotherapy, hormonal therapy, biological therapy, and photodynamic therapy.^1^

More recently, targeted therapy has shown great potential in addressing drugs toward cancer cells of specific genes and proteins without attacking the healthy cells. It is well known that protein kinases play a vital role in regulating cell function. Therefore, these proteins can be used as a molecular target for designing new cancer inhibitors. For example, it was found that most human cancers are associated with the deregulation of cyclin-dependent kinases (CDKs). CDKs are a family of serine-threonine kinases that regulate cell cycle progression *via* the phosphorylation process. CDKs play an essential role in the inactivation of the retinoblastoma tumor suppressor gene (Rb) and the G2/M damage checkpoint. However, designing CDK selective inhibitors is still the main drawback because the ATP-binding site of the CDKs is highly protected across the enzyme. CDK-2 is an S/T-protein kinase required for the cell cycle G1/S transition. The inhibition of CDK-2 modulates siRNA and generates cell cycle arrest and apoptosis, leading to decreased proliferation of several cancer cells. This class of enzymes has attracted great attention for the designing and preparation of selective cancer inhibitors. Several inhibitor-based CDK-2s have been developed and progressed into clinical evaluation, such as roscovitine, dinaciclib, and milciclib. Therefore, there is a clear need to design and synthesize novel, selective, and less-toxic bioactive antitumor agents.^2−4^

Recently, a molecular hybridization strategy based on incorporating two or more bioactive fragments into a single molecule has shown a simple, effective, and promising approach to discovering new drugs and could be beneficial for the treatment of cancer diseases.^5−7^

**Figure 1:**
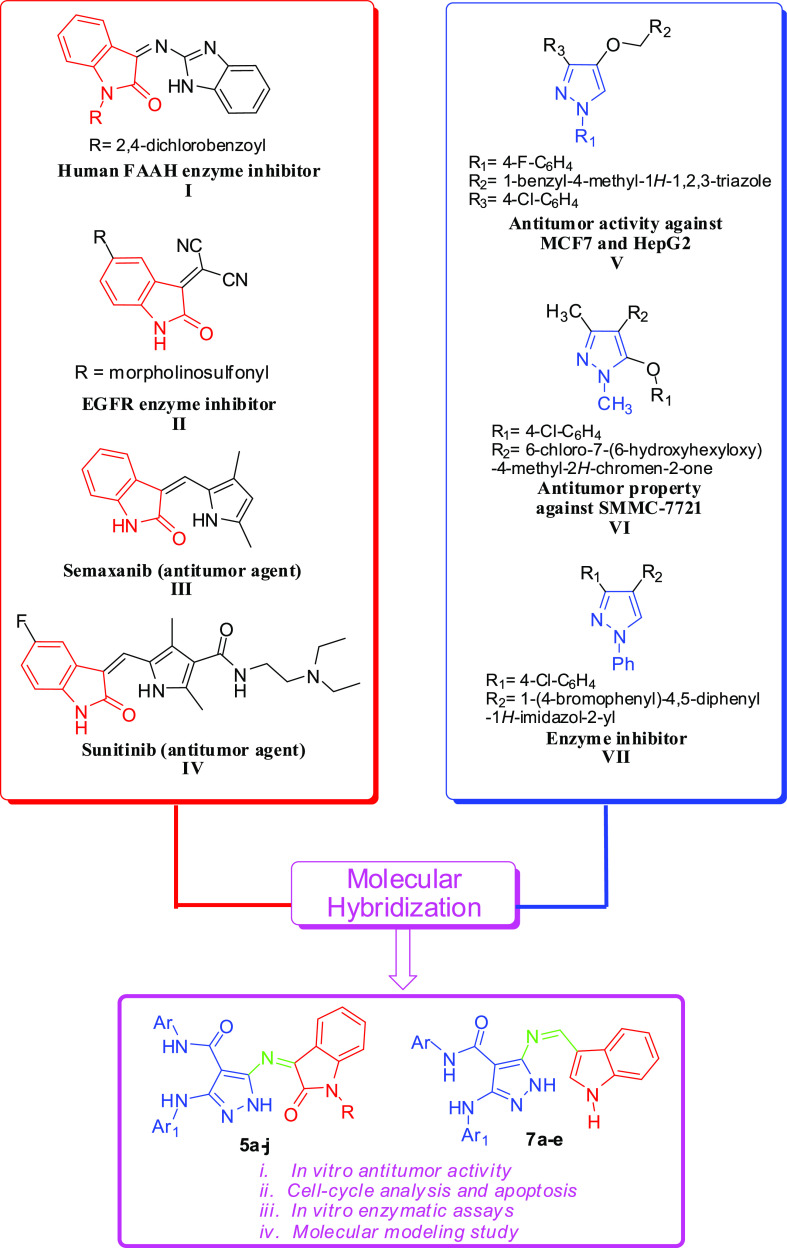
Schematic representation of the designed bioactive scaffold containing indole and pyrazole moieties (**5a–j** and **7a**–**e**).

In the last few decades, isatin (indoline-2,3-dione) derivatives have been widely used as a vital privileged scaffold in medical applications such as antitumor, antiviral, antimicrobial, antituberculosis, and enzyme inhibitors.^8−12^ 1*H*-Benzo[*d*]imidazol-2-ylimino-isatin (Figure 1I) showed an excellent inhibition performance against the human FAAH enzyme.^13^ Also, sulfonyl-isatin derivative afforded potent inhibitory activity against EGFR (Figure 1II).^14^Figure 1 presents some of the novel potential antitumor and cytotoxic agent-based indole derivatives, such as semaxanib (III) and sunitinib (IV).^15,16^

The pyrazole moiety displayed interesting biological activities for cancer treatment.^17−19^ For example, the pyrazole compound (Figure 1V) showed significant antitumor activity against the breast (MCF-7) and the liver (HepG2).^20^ 1,3-Dimethyl-1*H*-pyrazole derivative (Figure 1VI) demonstrated low acute toxicity and a potent antitumor property against SMMC-7721 cell line *in vivo*.^21^ Furthermore, pyrazole compounds play an essential role as potent enzyme inhibitors. For example, 1-phenyl-1*H*-pyrazole derivatives can be used as an inhibitor of α-glucosidase (Figure 1VII).^22^

Based on the aforementioned considerations and in continuation of our research program aimed to develop bioactive candidates,^23−44^ we have designed and synthesized a series of novel pyrazole–indole hybrids (**5a**–**j** and **7a**–**e**) and evaluated their anticancer activity *in vitro* against four human cancer cells [HCT-116, MCF-7, HepG2, and A549] using the MTT assay. Moreover, the two most potent target compounds (**7a** and **7b**) have been selected to investigate their mechanism of action (cell cycle analysis and apoptosis investigation), enzymatic assays against caspase-3, Bcl-2, Bax, and CDK-2 kinase enzymes. Besides, we have studied the molecular modeling for both chemicals to understand the interactions with the active site of the proteins. The schematic diagram of the design strategy of the new anticancer agents is depicted in Figure 1.

## 2 Results and Discussion

### 2.1 Chemistry

**Scheme 1:**
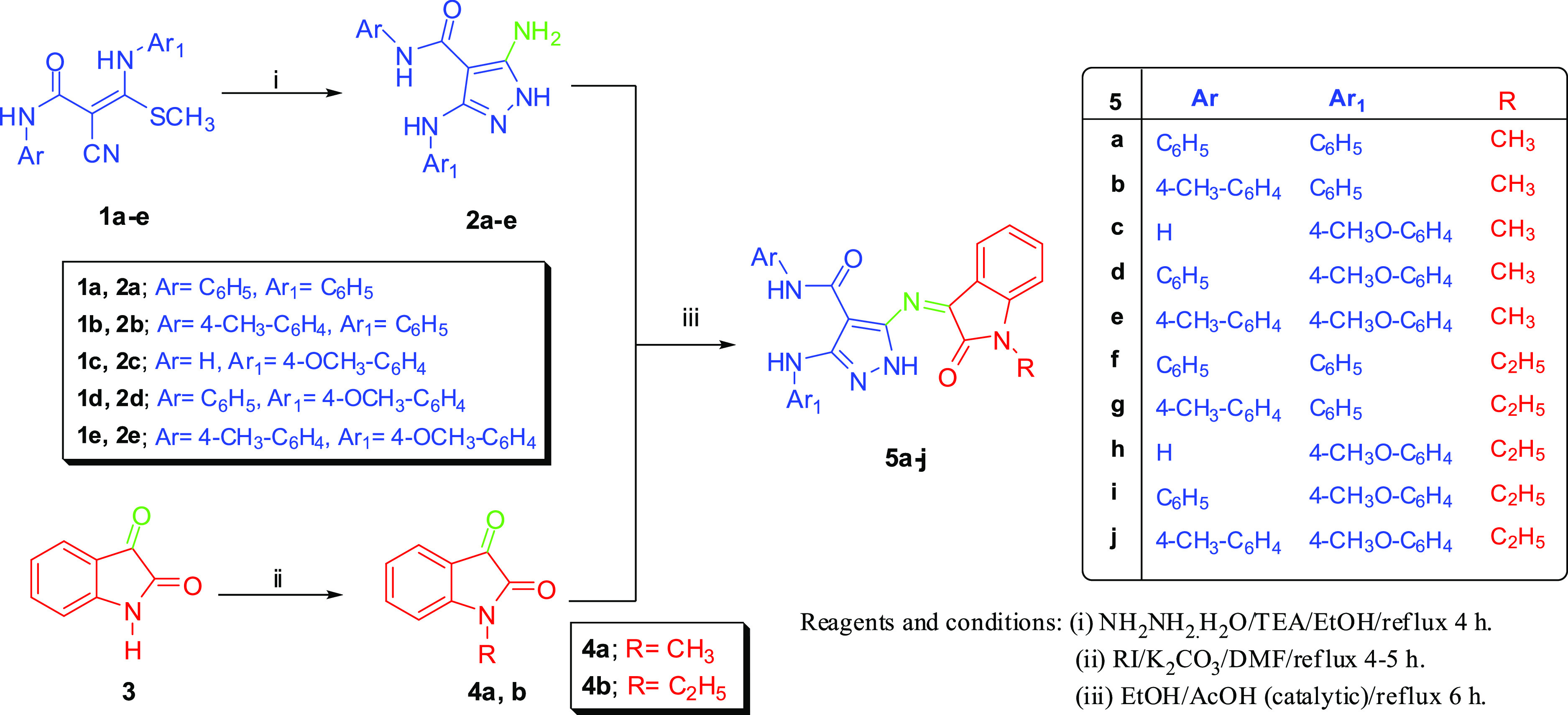
Schematic Representation of the Synthesis of Pyrazole–Oxindole Hybrids **5a**–**j**

**Scheme 2:**
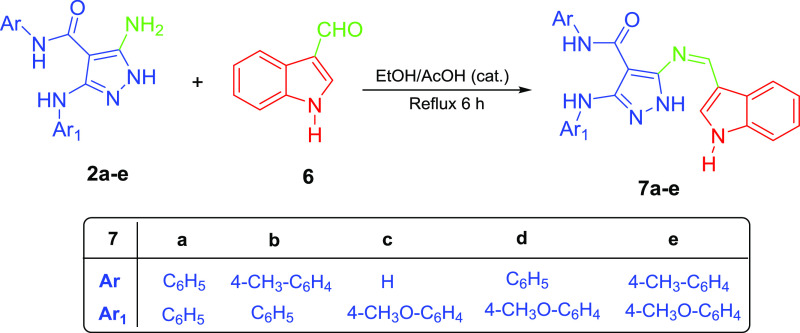
Synthesis of Pyrazole–Indole Hybrids **7a**–**e**

The synthetic pathways of new pyrazole–indole hybrids (**5a**–**j** and **7a**–**e**) are outlined in Schemes 1 and 2. The starting materials, 5-aminopyrazoles **2a**–**e**, were prepared by the reaction of *N*-aryl-3-(arylamino)-2-cyano-3-(methylthio)acrylamide derivative **1a**–**e** with hydrazine hydrate in refluxed ethanol in the presence of a catalytic amount of triethylamine.^45−48^ Also, *N*-substituted isatin **4a**,**b** were prepared by the reaction of isatin with alkyl iodide in dimethylformamide (DMF) in the presence of K_2_CO_3_.^49^ The target products, pyrazole–oxindole hybrids (**5a**–**j**), were prepared *via* the direct condensation of 5-aminopyrazoles **2a**–**e** with *N*-substituted isatin **4a**,**b** in refluxing EtOH in the presence of a catalytic amount of AcOH acid, as shown in Scheme 1.

The structure of pyrazole–oxindole hybrids **5a**–**j** was confirmed based on their spectral data. The ^1^H NMR spectrum of 5-(1-ethyl-2-oxoindolin-3-ylideneamino)-3-(4-methoxyphenylamino)-*N*-phenyl-1*H*-pyrazole-4-carboxamide (**5i**) revealed one triplet at δ 1.24 (3H, *J* = 7.2 and 7.1 Hz), one singlet signal at 3.76 (s, 3H), and one quartet at 3.85 (q, 2H, *J* = 7.2 Hz) attributed to methyl (−NCH_2_C**H**_**3**_), methoxy (−OC**H**_3_), and methylene (−NC**H**_**2**_CH_3_) protons, respectively. The three protons of 3NH appear as three signals at δ 8.81, 11.15, and 13.18 ppm. Furthermore, the nine protons of the two aromatic rings, phenyl (5H) and 4-methoxyphenyl (4H), appear as one doublet at 6.96 (2H, *J* = 8.8 Hz), one triplet at 7.06 (1H, *J* = 7.4 and 7.4 Hz), one doublet at 7.29 (2H, *J* = 8.6 Hz), one triplet at 7.36 (2H, *J* = 8.0 and 7.7 Hz), and one doublet at 7.95 (2H, *J* = 7.9 Hz), while the four protons of the isatin ring appear as one triplet at 7.12 (1H, *J* = 7.7 and 7.6 Hz), one doublet at 7.21 (1H, *J* = 7.8 Hz), one triplet at 7.58 (1H, *J* = 7.7 and 7.8 Hz), and one doublet at 9.15 (1H, *J* = 7.6 Hz). The ^13^C NMR spectrum of **5i** afforded five characteristic signals at δ 12.52, 34.46, 55.32, 162.28, and 163.52 ppm for the methyl, −NCH_2_, OCH_3_, C=O, and C=O carbon atoms, respectively.

Finally, 1*H*-indole-3-carbaldehyde (**6**) was refluxed with 5-aminopyrazoles **2a**–**e** in ethanol as a solvent and a catalytic amount of AcOH acid, giving pyrazole–indole hybrids **7a**–**e**, as shown in Scheme 2.

The structures of the target products **7a**–**e** were confirmed by spectroscopic techniques. For example, the ^1^H NMR spectrum of 5-((1*H*-indol-3-yl)methyleneamino)-3-(4-methoxyphenylamino)-*N*-(4-methylphenyl)-1*H*-pyrazole-4-carboxamide (**7e**) showed two signals at 2.26 and 3.72 for the protons of methyl (s, 3H, CH_3_) and methoxy (s, 3H, OCH_3_) groups, respectively. Three signals at 9.05 (1NH), 9.96 (1NH), and 12.26 (2NH) are assigned to the four NH protons. Two typical signals were observed at 8.31 and 8.78 for the protons of indole (1H) and the azomethine function (1H, −N=CH−), respectively. The protons of para-substitution phenyl rings (8H) appeared as four doublets at 6.89 (2H), 7.13 (2H), 7.48 (2H), and 7.53 (2H) with the coupling contents of 9.0 Hz, 8.3 Hz, 8.5 Hz, and 8.4 Hz, respectively. The four protons of the indole ring appeared as two triplets and two doublets. The two triplets were observed at 7.25 (1H, *J* = 7.8 and 7.9 Hz) and 7.34 (1H, *J* = 8.2 and 8.1 Hz), and the two doublets were found at 7.59 (1H, *J* = 8.1 Hz) and 8.34 (1H, *J* = 7.7 Hz).

Furthermore, the ^13^C NMR spectrum of the pyrazole–indole hybrid **7e** showed distinct signals of CH_3_ and OCH_3_ at 20.37 and 55.22 ppm, respectively. In addition, ^13^C NMR displayed a distinct singlet signal at 163.12 ppm corresponding to the C=O group.

### 2.2 Biological Evaluation

#### 2.2.1 *In Vitro* Anticancer Screening

Fifteen compounds (**5a**–**j** and **7a**–**e**) were examined *in vitro* for their activities on HCT-116, MCF-7, HepG2, and A549 human cancer cells using the MTT assay.^50−52^

The percentages of intact cells were calculated and compared to those of the control. Activities of these compounds against the four cell lines were compared to the activity of doxorubicin as well. All compounds suppressed the four human cells in a dose-dependent manner (Table 1). To study the efficacy of the synthesized compounds (**5a**–**j** and **7a**–**e**), a comparison of the cytotoxic effect of each compound has been related to the cytotoxicity of the reference drug as follows.

- In the case of HCT-116 human colorectal carcinoma cells, 11 compounds, **5a**, **5b**, **5f**, **5g**, **5h**, **5j**, **7a**, **7b**, **7c**, **7d**, and **7e**, with a range from IC_50_ = 17.4 ± 3.2 to 38.9 ± 4.1 μM, showed significantly more potent anticancer activities. Two compounds **5c** (IC_50_ = 39.5 ± 4.5 μM) and **5e** (IC_50_ = 39.9 ± 4.2 μM) showed an equipotent anticancer effect compared to doxorubicin. In addition, two compounds **5d** (IC_50_ = 54.2 ± 5.5 μM) and **5i** (IC_50_ = 53.9 ± 5.7 μM) afforded weak anticancer activities in comparison with doxorubicin (IC_50_ = 40.0 ± 3.9 μM).
- In the case of MCF-7 human breast cancer cells, all of the synthesized compounds (**5a**–**j** and **7a**–**e**) afforded excellent anticancer activities with a range from IC_50_ = 10.6 ± 2.3 to 63.7 ± 5.5 μM compared to doxorubicin (IC_50_ = 64.8 ± 4.1 μM).
- In the case of HepG2 human liver cancer cells, three compounds **5f** (IC_50_ = 23.7 ± 3.1 μM), **7a** (IC_50_ = 6.1 ± 1.9 μM), and **7b** (IC_50_ = 7.9 ± 1.9 μM) displayed potential inhibition performance compared to the cytotoxic reference compound [doxorubicin (IC_50_ = 24.7 ± 3.2 μM)]. Compound **5j** (IC_50_ = 24.4 ± 2.9 μM) showed equipotent activity. It was also found that the remainder of the tested compounds (**5a**–**e**, **5h**, **5i**, **7c**, **7d**, and **7e**) showed poor inhibition performance compared to doxorubicin (IC_50_ = 24.7 ± 3.2 μM).
- In the case of A549 human lung cancer cells, 13 compounds (**5a**–**d**, **5f**–**5j**, **7a**–**7c**, and **7e**) provided powerful anticancer activities. Compound **7d** (IC_50_ = 58.7 ± 4.2 μM) had equipotent activity. On the contrary, compound **5e** (IC_50_ = 77.7 ± 5.6 μM) exhibited low anticancer activity compared to doxorubicin (IC_50_ = 58.1 ± 4.1 μM).

**Table 1:**
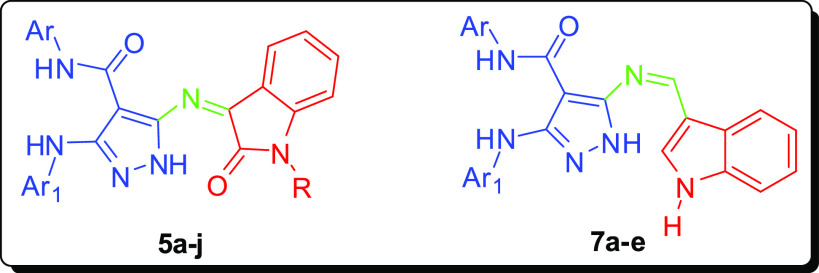
IC_50_ (μM) of the 15 Compounds (**5a**–**j** and **7a**–**e**) against the Four Cancer Cell Lines Using the MTT Assay

				**IC**_**50**_ **(μM) ± SD**			
**compounds**	**Ar**	**Ar**_**1**_	**R**	**HCT-116**	**MCF-7**	**HepG2**	**A549**
**5a**	C_6_H_5_	C_6_H_5_	CH_3_	25.7 ± 3.5	28.3 ± 3.5	27.6 ± 3.1	42.8 ± 4.2
**5b**	4-CH_3_-C_6_H_4_	C_6_H_5_	CH_3_	28.1 ± 3.5	48.0 ± 5.1	37.1 ± 4.3	46.7 ± 4.9
**5c**	H	4-CH_3_O-C_6_H_4_	CH_3_	39.5 ± 4.5	22.0 ± 3.5	32.8 ± 3.8	40.7 ± 4.1
**5d**	C_6_H_5_	4-CH_3_O-C_6_H_4_	CH_3_	54.2 ± 5.5	46.4 ± 4.5	34.8 ± 3.9	56.0 ± 4.1
**5e**	4-CH_3_-C_6_H_4_	4-CH_3_O-C_6_H_4_	CH_3_	39.9 ± 4.2	61.8 ± 5.1	32.0 ± 3.9	77.7 ± 5.6
**5f**	C_6_H_5_	C_6_H_5_	C_2_H_5_	28.9 ± 3.9	42.1 ± 4.7	23.7 ± 3.1	52.9 ± 4.5
**5g**	4-CH_3_-C_6_H_4_	C_6_H_5_	C_2_H_5_	35.7 ± 3.9	54.0 ± 4.9	27.8 ± 3.5	46.8 ± 5.3
**5h**	H	4-CH_3_O-C_6_H_4_	C_2_H_5_	25.7 ± 4.3	25.4 ± 3.9	28.2 ± 3.5	41.9 ± 3.9
**5i**	C_6_H_5_	4-CH_3_O-C_6_H_4_	C_2_H_5_	53.9 ± 5.7	30.1 ± 4.2	26.7 ± 3.1	47.8 ± 4.5
**5j**	4-CH_3_-C_6_H_4_	4-CH_3_O-C_6_H_4_	C_2_H_5_	38.9 ± 4.1	63.7 ± 5.5	24.4 ± 2.9	57.0 ± 4.9
**7a**^a^	C_6_H_5_	C_6_H_5_		17.4 ± 3.2	10.6 ± 2.3	6.1 ± 1.9	23.7 ± 3.1
**7b**^a^	4-CH_3_-C_6_H_4_	C_6_H_5_		19.6 ± 3.5	14.5 ± 2.5	7.9 ± 1.9	14.1 ± 2.1
**7c**	H	4-CH_3_O-C_6_H_4_		31.9 ± 3.8	22.2 ± 3.3	35.8 ± 3.9	43.4 ± 4.2
**7d**	C_6_H_5_	4-CH_3_O-C_6_H_4_		25.3 ± 3.5	17.4 ± 2.3	27.2 ± 3.5	58.7 ± 4.2
**7e**	4-CH_3_-C_6_H_4_	4-CH_3_O-C_6_H_4_		37.4 ± 4.1	16.2 ± 2.3	25.8 ± 3.5	40.8 ± 4.3
doxorubicin				40.0 ± 3.9	64.8 ± 4.1	24.7 ± 3.2	58.1 ± 4.1

a The most potent compound as new anticancer agents.

We can conclude from the above results that both compounds (**7a** and **7b**) showed an excellent cancer inhibition performance. They could be proposed as candidate drugs for human colon cancer, breast cancer, liver cancer, and lung cancer types.

#### 2.2.2 Cell Cycle Analysis and Apoptosis Detection

**Figure 2:**
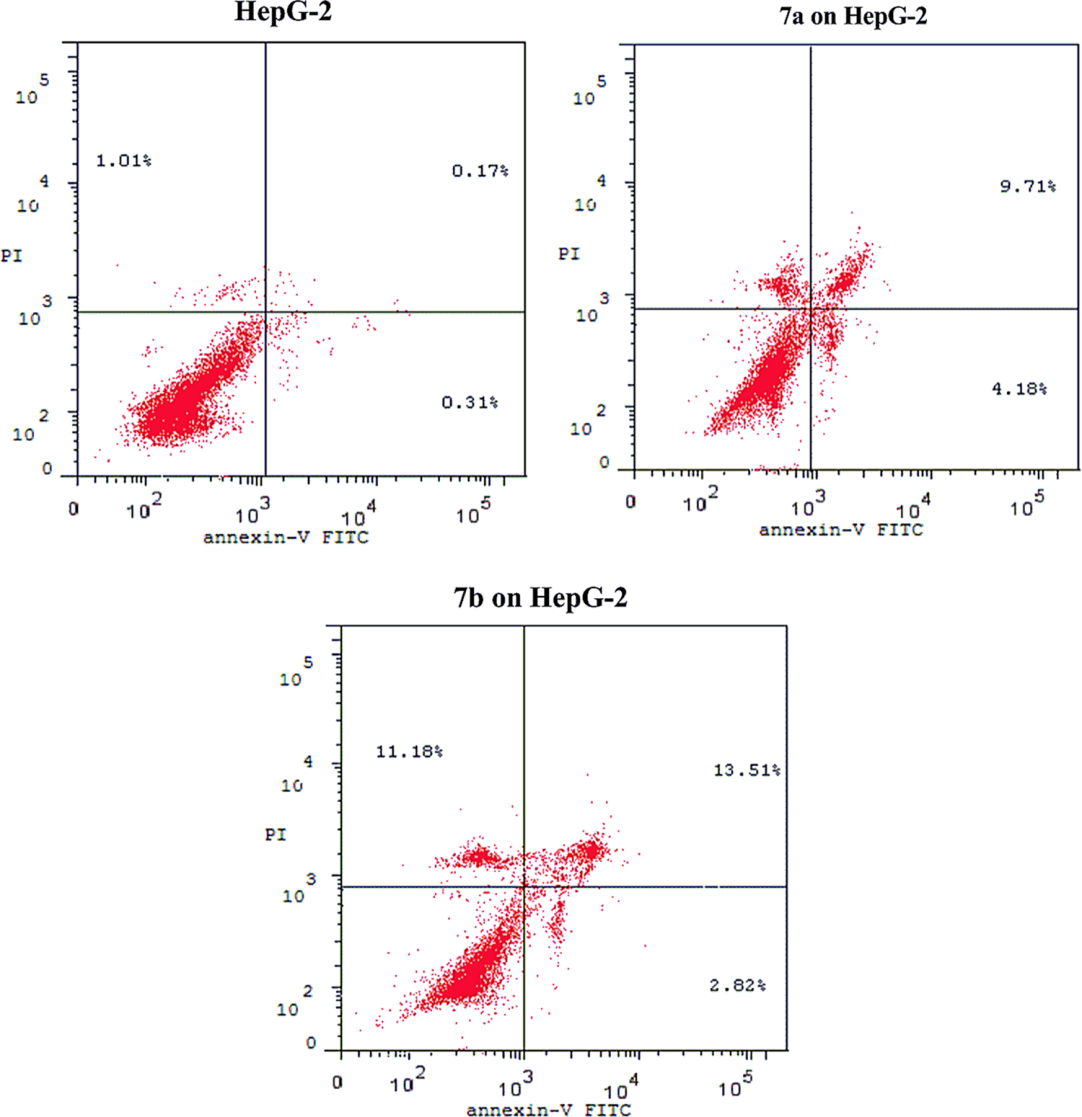
Flow cytometry apoptotic status on HepG2 cancer cells for the negative control, DMSO, and compounds (**7a**,**b**), respectively.

Compounds **7a** and **7b** showed the best cytotoxic activities compared to the commercial cytotoxic reference compound, as well as other synthesized pyrazole derivatives. These results encouraged us to study the cellular mechanistic action of both compounds on the progression of the cell cycle and induction of apoptosis on the HepG2 cell line. The induction of apoptosis has been investigated using the annexin V/propidium iodide (PI) staining assay for both compounds **7a** and **7b** on HepG2. It was found that compounds **7a** and **7b** induced more apoptotic cells (annexin V+/PI– and annexin V+/PI+), producing total necrosis and apoptosis (early and late) percentages of 22.18 and 27.51%, respectively, compared to the negative control dimethyl sulfoxide (DMSO) (1.49%), as presented in Figure 2.

**Figure 3:**
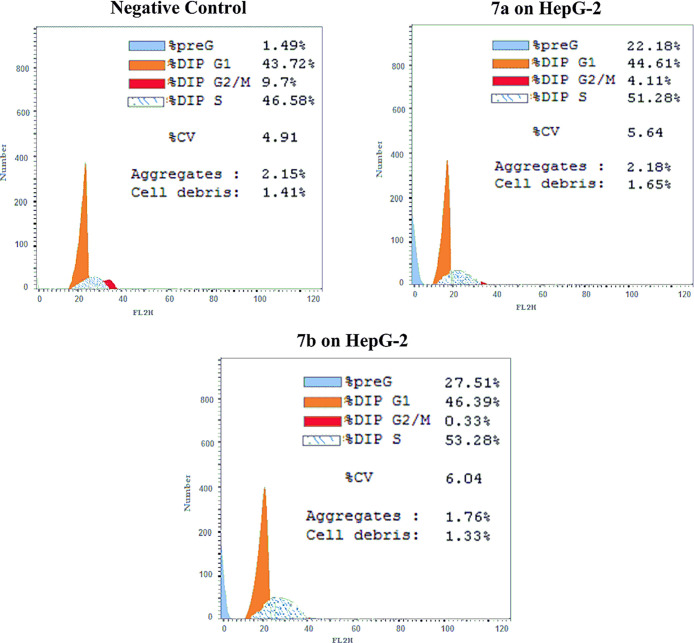
Flow cytometry cancer cell cycle distribution on HepG2 cancer cells for the negative control, DMSO, and compounds (**7a**,**b**), respectively.

To elucidate whether the cytotoxic activity is due to suppression of cell cycle progression, HepG2 cells were exposed to compounds **7a** and **7b** at concentrations of 7.9 and 6.1 μM, respectively, for 24 h and analyzed using flow cytometry. The obtained results revealed that compounds **7a** and **7b** induced significant accumulation of cells at the Pre G1 phase by 14.9- and 18.5-fold comparing to the control, showing a significant reduction in the percentage of cells at the G2/M phase by 2.4- and 29.4-fold, respectively. These compounds also provided a slight increase in S phases by 0.1- and 0.14-fold, respectively, compared to the reference control, as shown in Figure 3.

#### 2.2.3 Enzymatic Assay

##### 2.2.3.1 Effect of Compounds **7a** and **7b** on the Levels of Caspase-3, Bcl-2, and Bax

It has been reported that caspases cascade through either intrinsic or extrinsic pathways that mediate the induction of apoptosis, which may lead to apoptotic cell death.^53−55^ Caspase-3 is involved in cell shrinkage, chromatin condensation, and DNA fragmentation inside the cells, causing apoptosis induction. In this study, the bioluminescent intensities of caspase-3 for both compounds **7a** and **7b** indicated that caspase-3 activation has been measured in HepG2 cells, treated at concentrations of 7.9 and 6.1 μM, respectively, for 24 h. As shown in Table 2, a significant increase in caspase-3 activities was detected for both compounds **7a** and **7b** compared to the negative control. They showed 7- and 5.8-fold higher activation, respectively.

**Table 2:** Results of Caspase-3/Bax/BCL-2 Analysis after Treatment of Cells with Two Compounds **7a** and **7b**

**compounds**	**caspase-3** (Pg/mL)	**Bcl-2** (ng/mL)	**Bax** (Pg/mL)
**7a**	388.7 ± 7	3.123 ± 0.1	211.3 ± 5.9
**7b**	469.8 ± 10	2.479 ± 0.07	272.6 ± 11.5
cont. HepG2	67.3 ± 2.8	6.222 ± 0.14	5.762 ± 1.18

Furthermore, it is well known that the antiapoptotic Bcl-2 protein plays a critical role in cancer resistance therapy.^56^ Therefore, we have studied the effect of both compounds **7a** and **7b** on Bcl-2 protein expression levels. It was found that **7a** and **7b** caused significant downregulation of the Bcl-2 protein level, as tabulated in Table 2. They provided a 0.5- and 0.4-fold decrease in the Bcl-2 concentration, respectively. These results agree with the cell cycle and apoptosis results, which indicated that both compounds could induce apoptosis by cell cycle arrest and/or by inhibition of Bcl-2.

In addition, the pro-apoptotic protein (Bax) is a protein that accelerates apoptosis by binding to and antagonizing the death repressor activity of Bcl-2.^57^ Following any apoptotic stimuli, Bax causes activation of caspase-3 and perpetuates the apoptotic cascade.^58^ The Bax protein expression level is altered in various human malignancies.^59,60^ Therefore, the effect of both compounds **7a** and **7b** on the Bax expression level has been studied. The obtained results showed that both compounds **7a** and **7b** caused significant upregulation of the Bax protein level as they showed an 8.2- and 10.6-fold increase in the Bax concentration, respectively (Table 2).

Overall, the above results may indicate that the stimulation of the apoptotic pathway by both compounds **7a** and **7b** further affects the upregulation of Bax protein, leading to stimulation of caspase-3 upregulation and Bcl-2 downregulation.

##### 2.2.3.2 *In Vitro* CDK-2 Kinase Assessment

The promising antiproliferative impact of the conjugates **7a** and **7b**, besides their cell cycle disruption and pro-apoptotic effects, pushed for additional exploration for their inhibitory activities against the cell cycle regulator CDK-2 enzyme. Table 3 summarizes the inhibitory assessment (IC_50_) of compounds **7a** and **7b** compared to the reference control roscovitine. The analyzed results showed that compounds **7a** and **7b** demonstrated superior inhibitory activity toward CDK-2 in comparison with roscovitine (IC_50_ = 0.074 ± 0.15, 0.095 ± 0.10, and 0.100 ± 0.25 μM, respectively).

**Table 3:** Inhibitory Assessment (IC_50_ in μM) of Compounds **7a** and **7b** on CDK-2 Enzyme Performed Using Enzyme-Linked Immunosorbent Assay (ELISA)

**compounds**	**CDK-2 (IC**_**50**_**, μM)**
**7a**	0.074 ± 0.15
**7b**	0.095 ± 0.10
roscovitine	0.100 ± 0.25

### 2.3 Molecular Docking Study

This molecular docking study aims to understand the possible binding modes of the potential anticancer compounds **7a** and **7b** with the key amino acids (hot spots) in the active site of the CDK-2 enzyme. This study was performed using Molecular Operating Environment (MOE) 2008.10. The X-ray crystal structure of CDK-2 (PDB code: 2A4L)^61^ was downloaded from the Protein Data Bank.

**Figure 4:**
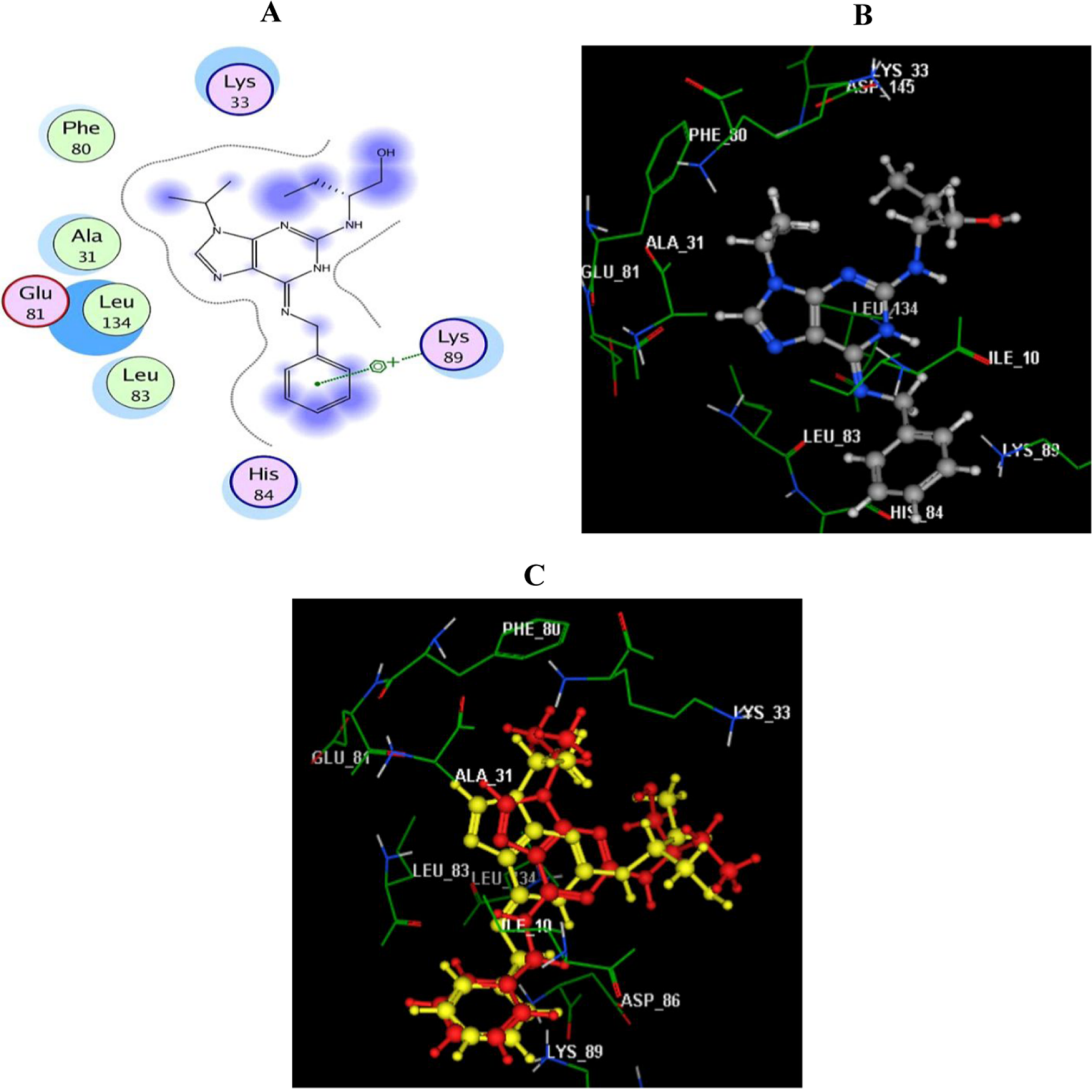
(A, B) Two-dimensional (2D) and three-dimensional (3D) images of the native ligand (roscovitine) redocked in the ATP active site of CDK-2 (PDB ID: 2A4L) using MOE software. (C) 3D image of the superimposition of the docking pose (yellow) and the co-crystallized inhibitor pose (red) of roscovitine with an RMSD of 0.72 Å.

Validation of the docking protocol was first performed by redocking of the co-crystallized ligand roscovitine in the CDK-2 active site. The redocking validation step confirmed that the docking protocol used is suitable for the subsequent docking study. This is illustrated by the score energy of −11.25 kcal/mol and the small root mean standard deviation (RMSD) between the docked pose and the co-crystallized inhibitor pose of 0.72 Å and the highly observed superimposition between them (Figure 4C). The benzyl moiety of the co-crystallized ligand (roscovitine) interacts with the active site of CDK-2 by arene–cation interaction with the essential amino acid Lys89. In addition, roscovitine formed many hydrophobic interactions with other amino acid residues, Ala31, Lys33, Phe80, Glu81, Leu83, His84, and Leu134, as shown in Figure 4A,B.

**Figure 5:**
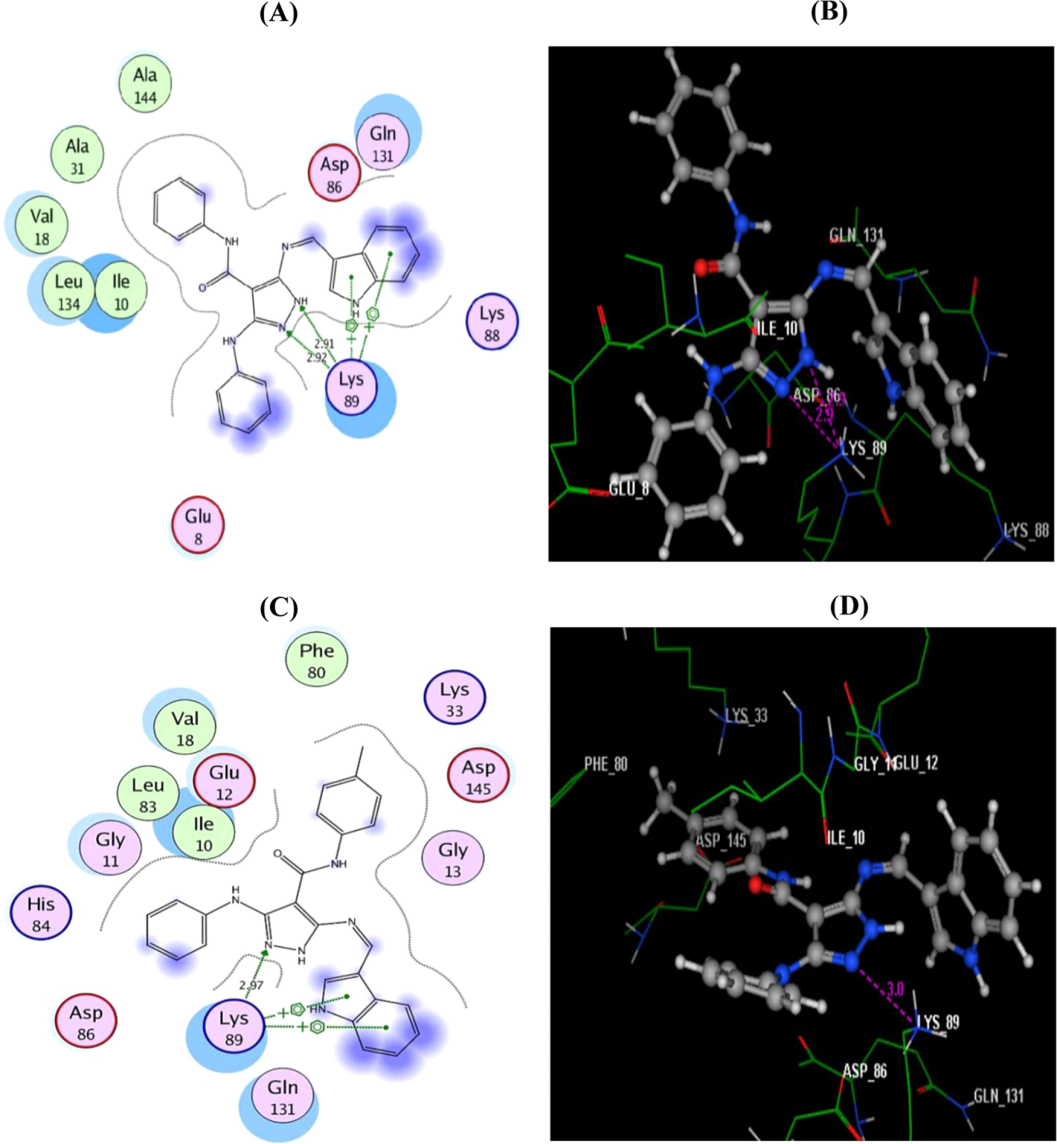
(A, B) Two-dimensional (2D) and three-dimensional (3D) interaction diagrams of docked compound **7a** with CDK-2 (PDB code: 2A4L). (C, D) 2D and 3D interaction diagrams of compound **7b** with CDK-2 (PDB code: 2A4L). Hydrogen bonds are illustrated as arrows. Carbon atoms are labeled in gray, nitrogen atoms in blue, and oxygen atoms in red.

Subsequently, the docking procedure for both compounds **7a** and **7b** was investigated, as shown in Figure 5. The corresponding 2D and 3D diagrams of the binding modes of both inhibitors with higher negative energy scores of −13.68 and −12.55 kcal/mol denote higher predicted binding affinity than that of the native ligand.

It was found that the docked derivatives **7a** and **7b** were fitted within the active site of the enzyme using the same crucial amino acid residue Lys89 *via* two arene–cation interactions with the centroids of indole and H-bonding with the N2 of the pyrazole moiety (distance: 2.92 and 2.97 Å, respectively). Upon investigation, it was also found that the N1 of pyrazole **7a** supported the binding through another hydrogen bond donor with the side chain of Lys89 (distance: 2.91 Å).

**Figure 6:**
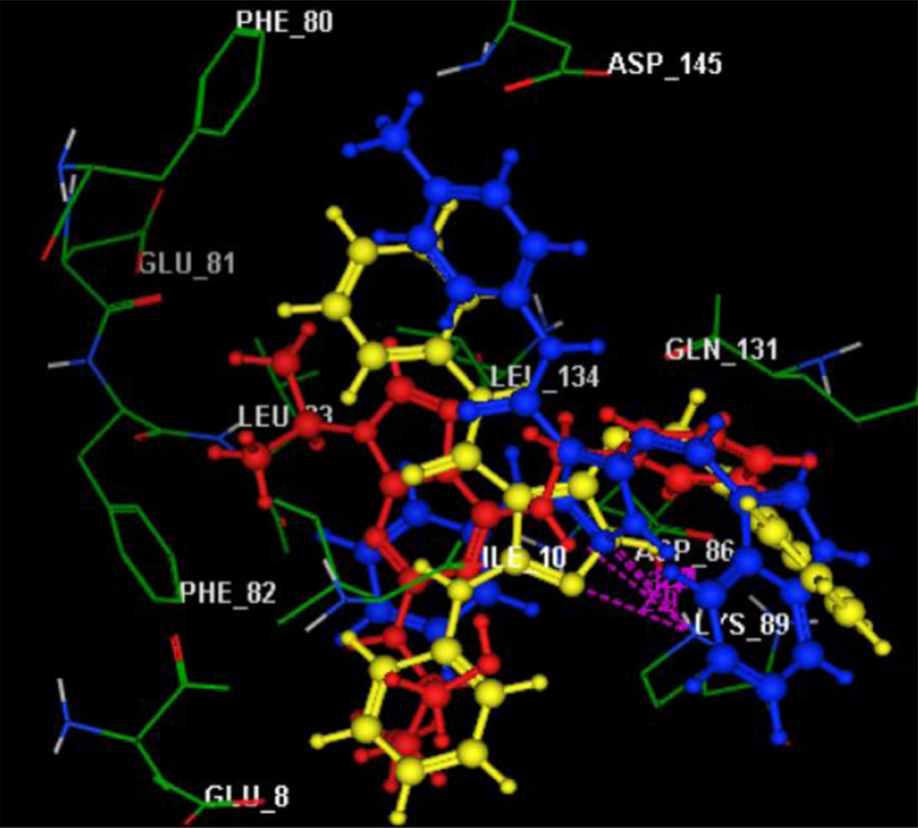
3D representation of docked roscovitine (red) in superimposition with compounds **7a** (yellow) and **7b** (blue) in the active site of CDK-2 (PDB code: 2A4L).

Finally, we anticipated that the two compounds (**7a** and **7b**), including indole and pyrazole moieties, could well embed in the active pocket of CDK-2 *via* different interactions with the key amino acid Lys89. This is confirmed by the superimposition phenomenon, as explained in Figure 6. Moreover, the achieved binding pattern explored the superior CDK-2 inhibitory activity of these compounds than the co-crystalized inhibitor (roscovitine).

## 3 Conclusions

In this study, we have designed and synthesized for the first time a series of novel pyrazole–indole hybrids *via* a molecular hybridization protocol as anticancer agents. The target compounds (**5a**–**j** and **7a**–**e**) were screened against four types of human cancers [HCT-116, MCF-7, HepG2, and A549] using the MTT assay. The antiproliferative activity results showed that most synthesized compounds showed a moderate-to-excellent inhibition performance compared to the standard reference drug, doxorubicin. Interestingly, compounds **7a** and **7b** incorporating pyrazole–indole itself, and not the oxindole ring, displayed powerful inhibition against HepG2 and MCF-7 cancer cell lines. Moreover, these two compounds demonstrated significant inhibitory activity toward cyclin-dependent kinase 2 (CDK-2). Also, cell cycle experiments for compounds **7a**,**b** revealed significant accumulation of cells at the Pre G1 phase, as well as a late apoptotic induction effect, as demonstrated from the annexin V FTIC study. These two compounds induced a significant increase in the caspase-3 activities, remarkable downregulation of the Bcl-2 protein level, and significant upregulation of the Bax protein level.

Finally, the obtained results were supported by a molecular docking study of these two compounds bearing indole and pyrazole moieties, which revealed that these two compounds could fit well and interact with the active pocket of CDK-2 *via* different interactions. Overall, the results indicate that both compounds **7a** and **7b** can be proposed as promising CDK-2 inhibitors and anticancer candidate drugs.

## 4 Experimental Section

### 4.1 Chemistry

All melting points were measured on a Gallenkamp melting point apparatus and are uncorrected. The IR spectra were recorded (KBr disk) on a Perkin Elmer 1650 FT-IR instrument. ^1^H NMR (400 MHz) and ^13^C NMR (100 MHz) spectra were recorded on a Varian spectrometer using DMSO-*d*_6_ as a solvent and TMS as an internal standard. Chemical shifts are reported in ppm. Mass spectra were recorded on a Varian MAT 112 spectrometer at 70 eV. Elemental analyses were performed at the Microanalytical Center, Cairo University, Egypt.

The progress of the reactions was monitored by thin-layer chromatography (TLC) using aluminum sheets coated with silica gel F_254_ (Merck, Darmstadt, Germany), with detection under ultraviolet light in the range of 254-360 nm. All evaporations were carried out under reduced pressure at 40 °C.

#### 4.1.1 General Procedure for the Preparation of Pyrazole–Oxindole Hybrids (**5a**–**j**)

A mixture of compounds **2a**–**e** (0.01 mol) and *N*-substituted isatin **4a**,**b** (0.01 mol) {namely, 1-methylindoline-2,3-dione (**4a**) and 1-ethylindoline-2,3-dione (**4b**)} with a catalytic amount of glacial acetic acid (0.5 mL) in absolute ethanol (25 mL) was refluxed for 1 h and then left to cool. The solid product was filtered off, dried, and finally recrystallized from ethanol to afford target products **5a**–**j**.

##### 4.1.1.1 5-(1-Methyl-2-oxoindolin-3-ylideneamino)-*N*-phenyl-3-(phenylamino)-1*H*-pyrazole-4-carboxamide (**5a**)

Black crystals, mp 270–272 °C, yield (74%). ^1^H NMR (DMSO-*d*_6_, 400 MHz, δ ppm): 3.25 (s, 3H, NCH_3_), 6.85 (t, 1H, *J* = 7.0 and 6.8 Hz, ArH), 7.04–7.15 (m, 4H, ArH), 7.34–7.38 (m, 4H, ArH), 7.58 (d, 2H, *J* = 7.7 and 7.7 Hz, ArH), 7.93 (d, 2H, *J* = 6.4 Hz, ArH), 8.99 (s, 1H, NH), 9.11 (d, 1H, *J* = 7.3 Hz, ArH), 11.16 (s, 1H, NH), 13.43 (s, 1H, NH). ^13^C NMR (DMSO-*d*_6_, 100 MHz, δ ppm): 26.30 (C, NCH_3_), 95.28, 109.75, 116.55, 118.31, 118.56, 122.41, 122.97, 128.89, 129.01, 129.50, 135.77, 138.13, 139.10, 147.94, 148.99, 158.86, 159.01, 162.14 (22C), 162.29, 163.81 (2C, 2C=O). Anal. calcd. (%) for C_25_H_20_N_6_O_2_ (436.47): C, 68.80; H, 4.62; N, 19.25. Found: C, 68.92; H, 4.55; N, 19.30%.

##### 4.1.1.2 5-(1-Methyl-2-oxoindolin-3-ylideneamino)-3-(phenylamino)-*N*-(4-methylphenyl)-1*H*-pyrazole-4-carboxamide (**5b**)

Brown crystals, mp > 300 °C, yield (70%). ^1^H NMR (DMSO-*d*_6_, 400 MHz, δ ppm): 2.27 (s, 3H, CH_3_), 3.25 (s, 3H, NCH_3_), 7.05 (t, 1H, *J* = 6.8 and 6 Hz, ArH), 7.10–7.16 (m, 4H, ArH), 7.30–7.38 (m, 4H, ArH), 7.59 (t, 1H, *J* = 7.6 and 6.5 Hz, ArH), 7.82 (d, 2H, *J* = 6.0 Hz, ArH), 9.06 (s, 1H, NH), 9.11 (d, 1H, *J* = 7.2 Hz, ArH), 11.07 (s, 1H, NH), 13.44 (s, 1H, NH). ^13^C NMR (DMSO-*d*_6_, 100 MHz, δ ppm): 20.47 (C, CH_3_), 26.30 (C, NCH_3_), 96.52, 109.73, 118.29, 118.50, 119.53, 122.41, 129.27, 129.39, 129.51, 131.86, 135.75, 136.62, 139.53, 147.16, 148.99, 153.11, 153.51 (22C), 161.96, 162.75 (2C, 2C=O). Anal. calcd. (%) for C_26_H_22_N_6_O_2_ (450.49): C, 69.32; H, 4.92; N, 18.66. Found: C, 69.25; H, 5.00; N, 18.60%.

##### 4.1.1.3 3-(4-Methoxyphenylamino)-5-(1-methyl-2-oxoindolin-3-ylideneamino)-1*H*-pyrazole-4-carboxamide (**5c**)

Dark brown crystals, mp 265–267 °C, yield (71%). ^1^H NMR (DMSO-*d*_6_, 400 MHz, δ ppm): 3.24 (s, 3H, NCH_3_), 3.74 (s, 3H, OCH_3_), 6.87 (d, 2H, *J* = 8.9 Hz, ArH), 7.06 (t, 1H, *J* = 7.6 and 7.5 Hz, ArH), 7.13 (d, 2H, *J* = 7.9 Hz, ArH), 7.26, 7.38 (2s, 2H, NH_2_), 7.48 (t, 1H, *J* = 7.7 and 7.9 Hz, ArH), 7.57 (d, 1H, *J* = 7.8 Hz, ArH), 8.90 (s, 1H, NH), 8.98 (d, 1H, *J* = 7.7 Hz, ArH), 12.96 (s, 1H, NH). ^13^C NMR (DMSO-*d*_6_, 100 MHz, δ ppm): 26.19 (C, NCH_3_), 55.29 (C, OCH_3_), 94.92, 110.05, 114.26, 114.67, 121.23, 122.38, 123.44, 134.92, 141.56, 146.25, 148.65, 152.97, 154.93, 159.42 (16C), 163.24, 163.36 (2C, 2C=O). Anal. calcd. (%) for C_20_H_18_N_6_O_3_ (390.40): C, 61.53; H, 4.65; N, 21.53. Found: C, 61.60; H, 4.58; N, 21.45%.

##### 4.1.1.4 3-(4-Methoxyphenylamino)-5-(1-methyl-2-oxoindolin-3-ylideneamino)-*N*-phenyl-1*H*-pyrazole-4-carboxamide (**5d**)

Dark green crystals, mp 271–273 °C, yield (74%). ^1^H NMR (DMSO-*d*_6_, 400 MHz, δ ppm): 3.24 (s, 3H, NCH_3_), 3.76 (s, 3H, OCH_3_), 6.96 (d, 2H, *J* = 8.8 Hz, ArH), 7.05 (t, 1H, *J* = 7.3 and 8.8 Hz, ArH), 7.11 (t, 1H, *J* = 6.6 and 7.0 Hz, ArH), 7.13 (d, 1H, *J* = 7.1 Hz, ArH), 7.28 (d, 2H, *J* = 8.6 Hz, ArH), 7.35 (t, 2H, *J* = 8.2 and 7.9 Hz, ArH), 7.56 (t, 1H, *J* = 7.8 and 7.7 Hz, ArH), 7.94 (d, 2H, *J* = 7.8 Hz, ArH), 8.80 (s, 1H, NH), 9.10 (d, 1H, *J* = 7.5 Hz, ArH), 11.12 (s, 1H, NH), 13.15 (s, 1H, NH). ^13^C NMR (DMSO-*d*_6_, 100 MHz, δ ppm): 26.27 (C, NCH_3_), 55.31 (C, OCH_3_), 94.17, 109.65, 114.74, 116.60, 118.44, 121.80, 122.34, 122.81, 128.87, 129.34, 132.05, 135.62, 139.27, 148.56, 148.91, 149.45, 149.57, 155.66 (22C), 162.27, 163.88 (2C, 2C=O). Anal. calcd. (%) for C_26_H_22_N_6_O_3_ (466.49): C, 66.94; H, 4.75; N, 18.02. Found: C, 67.00; H, 4.70; N, 18.00%.

##### 4.1.1.5 3-(4-Methoxyphenylamino)-5-(1-methyl-2-oxoindolin-3-ylideneamino)-*N*-(4-methylphenyl)-1*H*-pyrazole-4-carboxamide (**5e**)

Dark green crystals, mp 262–263 °C, yield (76%). ^1^H NMR (DMSO-*d*_6_, 400 MHz, δ ppm): 2.27 (s, 3H, CH_3_), 3.25 (s, 3H, NCH_3_), 3.76 (s, 3H, OCH_3_), 6.96 (d, 2H, *J* = 8.9 Hz, ArH), 7.09–7.16 (m, 4H, ArH), 7.28 (d, 2H, *J* = 8.2 Hz, ArH), 7.58 (t, 1H, *J* = 7.8 and 7.7 Hz, ArH), 7.82 (d, 2H, *J* = 7.5 Hz, ArH), 8.81 (s, 1H, NH), 9.10 (d, 1H, *J* = 6.7 Hz, ArH), 11.03 (s, 1H, NH), 13.15 (s, 1H, NH). ^13^C NMR (DMSO-*d*_6_, 100 MHz, δ ppm): 20.47 (C, CH_3_), 26.29 (C, NCH_3_), 55.32 (C, OCH_3_), 94.20, 109.68, 114.74, 116.60, 118.41, 119.45, 121.67, 122.36, 129.26, 131.70, 132.16, 135.63, 136.75, 148.50, 148.91, 149.39, 149.53, 155.48 (22C), 162.10, 163.86 (2C, 2C=O). MS (*m*/*z*, %): 480 (M^+^, 100). Anal. calcd. (%) for C_27_H_24_N_6_O_3_ (480.52): C, 67.49; H, 5.03; N, 17.49. Found: C, 67.40; H, 5.10; N, 17.55%.

##### 4.1.1.6 5-(1-Ethyl-2-oxoindolin-3-ylideneamino)-*N*-phenyl-3-(phenylamino)-1*H*-pyrazole-4-carboxamide (**5f**)

Dark brown crystals, mp 274–276 °C, yield (73%). ^1^H NMR (DMSO-*d*_6_, 400 MHz, δ ppm): 1.24 (t, 3H, *J* = 7.2 and 7.1 Hz, CH_2_***CH***_***3***_), 3.83 (q, 2H, *J* = 7.2 Hz, ***CH***_***2***_CH_3_), 6.86 (t, 1H, *J* = 7.6 and 6.6 Hz, ArH), 7.03–7.14 (m, 3H, ArH), 7.19 (d, 1H, *J* = 7.8 Hz, ArH), 7.24 (t, 1H, *J* = 8.3 and 7.3 Hz, ArH), 7.35–7.39 (m, 4H, ArH), 7.57 (t, 1H, *J* = 7.7 and 7.7 Hz, ArH), 7.93 (d, 2H, *J* = 7.7 Hz, ArH), 9.05 (s, 1H, NH), 9.10 (d, 1H, *J* = 7.2 Hz, ArH), 11.98 (s, 2H, 2NH). ^13^C NMR (DMSO-*d*_6_, 100 MHz, δ ppm): 14.10 (C, CH_3_), 34.50 (C, NCH_2_), 95.27, 109.80, 116.72, 118.31, 119.60, 122.36, 123.02, 128.93, 129.54, 130.93, 135.83, 139.13, 141.54, 147.99, 148.44, 158.98, 159.81, 162.21 (22C), 162.60, 163.41 (2C, 2C=O). Anal. calcd. (%) for C_26_H_22_N_6_O_2_ (450.49): C, 69.32; H, 4.92; N, 18.66. Found: C, 69.25; H, 5.00; N, 18.60%.

##### 4.1.1.7 5-(1-Ethyl-2-oxoindolin-3-ylideneamino)-3-(phenylamino)-*N*-(4-methylphenyl)-1*H*-pyrazole-4-carboxamide (**5g**)

Black crystals, mp 288–290 °C, yield (71%). ^1^H NMR (DMSO-*d*_6_, 400 MHz, δ ppm): 1.24 (t, 3H, *J* = 7.2 and 7.1 Hz, CH_2_***CH***_***3***_), 2.27 (s, 3H, CH_3_), 3.83 (q, 2H, *J* = 7.2 Hz, ***CH***_***2***_CH_3_), 7.06–7.38 (m, 8H, ArH), 7.59 (t, 2H, *J* = 6.4 and 7.7 Hz, ArH), 7.83 (d, 2H, *J* = 7.6 Hz, ArH), 9.07 (s, 1H, NH), 9.15 (d, 1H, *J* = 7.2 Hz, ArH), 11.09 (s, 1H, NH), 13.45 (s, 1H, NH). ^13^C NMR (DMSO-*d*_6_, 100 MHz, δ ppm): 12.51 (C, CH_3_), 20.47 (C, CH_3_), 34.45 (C, NCH_2_), 95.40, 109.73, 118.48, 119.51, 122.49, 128.92, 129.27, 129.53, 131.83, 135.74, 136.65, 139.50, 141.10, 147.07, 147.95, 159.00, 159.86, 161.97 (22C), 162.12, 163.46 (2C, 2C=O). Anal. calcd. (%) for C_27_H_24_N_6_O_2_ (464.52): C, 69.81; H, 5.21; N, 18.09. Found: C, 69.75; H, 5.27; N, 18.00%.

##### 4.1.1.8 5-(1-Ethyl-2-oxoindolin-3-ylideneamino)-3-(4-methoxyphenylamino)-1*H*-pyrazole-4-carboxamide (**5h**)

Dark green crystals, mp 269–271 °C, yield (75%). ^1^H NMR (DMSO-*d*_6_, 400 MHz, δ ppm): 1.21 (t, 3H, *J* = 6.9 and 6.5 Hz, CH_2_***CH***_***3***_), 3.71 (s, 3H, OCH_3_), 3.78 (q, 2H, *J* = 7.0 Hz, ***CH***_***2***_CH_3_), 6.87 (d, 2H, *J* = 8.7 Hz, ArH), 7.06 (t, 1H, *J* = 6.8 and 6.1 Hz, ArH), 7.21 (d, 2H, *J* = 8.8 Hz, ArH), 7.47 (s, 2H, NH_2_), 7.54 (t, 1H, *J* = 8.0 and 8.0 Hz, ArH), 7.75 (d, 1H, *J* = 7.4 Hz, ArH), 8.90 (s, 1H, NH), 8.98 (d, 1H, *J* = 7.6 Hz, ArH), 13.00 (s, 1H, NH). ^13^C NMR (DMSO-*d*_6_, 100 MHz, δ ppm): 12.47 (C, CH_3_), 34.36 (C, NCH_2_), 55.30 (C, OCH_3_), 94.90, 109.64, 114.77, 117.52, 121.23, 123.30, 128.97, 132.35, 135.40, 139.68, 145.26, 148.34, 150.58, 155.42 (16C), 162.51, 163.07 (2C, 2C=O). MS (*m*/*z*, %): 404 (M^+^, 11.16). Anal. calcd. (%) for C_21_H_20_N_6_O_3_ (404.42): C, 62.37; H, 4.98; N, 20.78. Found: C, 62.30; H, 5.00; N, 20.70%.

##### 4.1.1.9 5-(1-Ethyl-2-oxoindolin-3-ylideneamino)-3-(4-methoxyphenylamino)-*N*-phenyl-1*H*-pyrazole-4-carboxamide (**5i**)

Dark brown crystals, mp 269–271 °C, yield (77%). ^1^H NMR (DMSO-*d*_6_, 400 MHz, δ ppm): 1.24 (t, 3H, *J* = 7.2 and 7.1 Hz, CH_2_***CH***_***3***_), 3.76 (s, 3H, OCH_3_), 3.85 (q, 2H, *J* = 7.2 Hz, ***CH***_***2***_CH_3_), 6.96 (d, 2H, *J* = 8.8 Hz, ArH), 7.06 (t, 1H, *J* = 7.4 and 7.4 Hz, ArH), 7.12 (t, 1H, *J* = 7.7 and 7.6 Hz, ArH), 7.21 (d, 1H, *J* = 7.8 Hz, ArH), 7.29 (d, 2H, *J* = 8.6 Hz, ArH), 7.36 (t, 2H, *J* = 8.0 and 7.7 Hz, ArH), 7.58 (t, 1H, *J* = 7.7 and 7.8 Hz, ArH), 7.95 (d, 2H, *J* = 7.9 Hz, ArH), 8.81 (s, 1H, NH), 9.15 (d, 1H, *J* = 7.6 Hz, ArH), 11.15 (s, 1H, NH), 13.18 (s, 1H, NH). ^13^C NMR (DMSO-*d*_6_, 100 MHz, δ ppm): 12.52 (C, CH_3_), 34.46 (C, NCH_2_), 55.32 (C, OCH_3_), 94.19, 109.75, 114.76, 116.75, 181.45, 121.88, 122.30, 122.84, 128.90, 129.63, 132.04, 135.73, 139.25, 147.92, 148.63, 149.48, 149.63, 155.69 (22C), 162.28, 163.52 (2C, 2C=O). MS (*m*/*z*, %): 480 (M^+^, 13.67). Anal. calcd. (%) for C_27_H_24_N_6_O_3_ (480.52): C, 67.49; H, 5.03; N, 17.49. Found: C, 67.40; H, 5.10; N, 17.55%.

##### 4.1.1.10 5-(1-Ethyl-2-oxoindolin-3-ylideneamino)-3-(4-methoxyphenylamino)-*N*-(4-methylphenyl)-1*H*-pyrazole-4-carboxamide (**5j**)

Dark brown crystals, mp 240–241 °C, yield (79%). ^1^H NMR (DMSO-*d*_6_, 400 MHz, δ ppm): 1.24 (t, 3H, *J* = 7.2 and 7.1 Hz, CH_2_***CH***_***3***_), 2.27 (s, 3H, CH_3_), 3.76 (s, 3H, OCH_3_), 3.84 (q, 2H, *J* = 7.1 Hz, ***CH***_***2***_CH_3_), 6.95 (d, 2H, *J* = 8.8 Hz, ArH), 7.11 (t, 1H, *J* = 7.6 and 7.8 Hz, ArH), 7.15 (d, 2H, *J* = 8.3 Hz, ArH), 7.20 (d, 1H, *J* = 7.9 Hz, ArH), 7.27 (d, 2H, *J* = 8.6 Hz, ArH), 7.57 (t, 1H, *J* = 7.7 and 7.7 Hz, ArH), 7.83 (d, 2H, *J* = 8.0 Hz, ArH), 8.82 (s, 1H, NH), 9.14 (d, 1H, *J* = 7.6 Hz, ArH), 11.05 (s, 1H, NH), 13.17 (s, 1H, NH). ^13^C NMR (DMSO-*d*_6_, 100 MHz, δ ppm): 12.52 (C, CH_3_), 20.47 (C, CH_3_), 34.45 (C, NCH_2_), 55.31 (C, OCH_3_), 94.24, 109.71, 114.75, 116.76, 181.41, 121.72, 122.27, 129.27, 129.62, 131.68, 132.10, 135.67, 136.76, 147.89, 148.50, 149.46, 149.57, 155.63 (22C), 162.12, 163.50 (2C, 2C=O). MS (*m*/*z*, %): 493 (M^+^ – 1, 45.05), 494 (M^+^, 9.75), 273 (100). Anal. calcd. (%) for C_28_H_26_N_6_O_3_ (494.54): C, 68.00; H, 5.30; N, 16.99. Found: C, 68.10; H, 5.25; N, 16.90%.

#### 4.1.2 General Procedure for the Preparation of 5-((1*H*-Indol-3-yl)methyleneamino)-*N*-aryl-3-(arylamino)-1*H*-pyrazole-4-carboxamide (**7a**–**e**)

A mixture of compounds **2a**–**e** (0.01 mol) and 1*H*-indole-3-carbaldehyde **6** (0.01 mol, 1.45 g) with a catalytic amount of glacial acetic acid (0.5 mL) in absolute ethanol (25 mL) was refluxed for 1 h and then left to cool. The solid product was filtered off, dried, and finally recrystallized from ethanol to afford compounds **7a**–**e**.

##### 4.1.2.1 5-((1*H*-Indol-3-yl)methyleneamino)-*N*-phenyl-3-(phenylamino)-1*H*-pyrazole-4-carboxamide (**7a**)

Yellow crystals, mp 266–268 °C, yield (73%). ^1^H NMR (DMSO-*d*_6_, 400 MHz, δ ppm): 6.87 (t, 1H, *J* = 7.3 and 7.3 Hz, ArH), 7.07 (t, 1H, *J* = 7.4 and 7.4 Hz, ArH), 7.24–7.37 (m, 6H, ArH), 7.57–7.65 (m, 5H, ArH), 8.33 (s, 1H, indole), 8.35 (d, 1H, *J* = 7.9 Hz, ArH), 9.00 (s, 1H, −N=CH−), 9.06 (s, 1H, NH), 10.03 (s, 1H, NH), 12.30 (s, 2H, 2NH). ^13^C NMR (DMSO-*d*_6_, 100 MHz, δ ppm): 92.05, 112.99, 114.34, 116.44, 119.42, 119.82, 121.23, 122.07, 123.33, 123.90, 124.35, 128.63, 128.98, 129.02, 137.73, 138.57, 141.36, 149.74, 152.03, 158.89 (24C), 163.29 (C, C=O). Anal. calcd. (%) for C_25_H_20_N_6_O (420.47): C, 71.41; H, 4.79; N, 19.99. Found: C, 71.35; H, 4.85; N, 20.05%.

##### 4.1.2.2 5-((1*H*-Indol-3-yl)methyleneamino)-3-(phenylamino)-*N*-(4-methylphenyl)-1*H*-pyrazole-4-carboxamide (**7b**)

Yellow crystals, mp 276–278 °C, yield (75%). ^1^H NMR (DMSO-*d*_6_, 400 MHz, δ ppm): 2.27 (s, 3H, CH_3_), 6.87 (t, 1H, *J* = 7.3 and 7.3 Hz, ArH), 7.14 (d, 2H, *J* = 8.3 Hz, ArH), 7.24–7.31 (m, 3H, ArH), 7.35 (t, 1H, *J* = 7.1 and 7.0 Hz, ArH), 7.53 (d, 2H, *J* = 8.4 Hz, ArH), 7.57 (d, 2H, *J* = 8.2 Hz, ArH), 7.60 (d, 1H, *J* = 8.1 Hz, ArH), 8.33 (s, 1H, indole), 8.34 (d, 1H, *J* = 7.6 Hz, ArH), 9.00 (s, 1H, −N=CH−), 9.05 (s, 1H, NH), 9.96 (s, 1H, NH), 12.29 (s, 2H, 2NH). ^13^C NMR (DMSO-*d*_6_, 100 MHz, δ ppm): 20.38 (C, CH_3_), 92.02, 112.94, 114.28, 116.34, 119.35, 121.18, 122.02, 123.84, 124.27, 128.98, 129.31, 132.27, 135.99, 137.66, 139.39, 141.34, 149.44, 153.15, 155.63, 158.66 (24C), 163.09 (C, C=O). Anal. calcd. (%) for C_26_H_22_N_6_O (434.49): C, 71.87; H, 5.10; N, 19.34. Found: C, 71.95; H, 5.00; N, 19.40%.

##### 4.1.2.3 5-((1*H*-Indol-3-yl)methyleneamino)-3-(4-methoxyphenylamino)-1*H*-pyrazole-4-carboxamide (**7c**)

Buff crystals, mp 260–262 °C, yield (75%). ^1^H NMR (DMSO-*d*_6_, 400 MHz, δ ppm): 3.71 (s, 3H, OCH_3_), 6.86 (d, 2H, *J* = 8.6 Hz, ArH), 7.23–7.56 (m, 7H, NH_2_ + ArH), 8.14 (d, 1H, *J* = 7.7 Hz, ArH), 8.24 (s, 1H, indole), 8.85 (s, 1H, −N=CH−), 8.94 (s, 1H, NH), 12.18 (s, 1H, NH), 12.38 (s, H, NH). ^13^C NMR (DMSO-*d*_6_, 100 MHz, δ ppm): 55.21 (C, OCH_3_), 86.37, 112.72, 114.30, 117.06, 120.77, 121.98, 123.55, 124.31, 128.16, 131.61, 134.87, 137.56, 147.18, 153.04, 158.45, 163.70 (18C), 166.70 (C, C=O). Anal. calcd. (%) for C_20_H_18_N_6_O_2_ (374.40): C, 64.16; H, 4.85; N, 22.45. Found: C, 64.10; H, 4.90; N, 22.39%.

##### 4.1.2.4 5-((1*H*-Indol-3-yl)methyleneamino)-3-(4-methoxyphenylamino)-*N*-phenyl-1*H*-pyrazole-4-carboxamide (**7d**)

Yellow crystals, mp 272–274 °C, yield (77%). ^1^H NMR (DMSO-*d*_6_, 400 MHz, δ ppm): 3.73 (s, 3H, OCH_3_), 6.90 (d, 2H, *J* = 8.2 Hz, ArH), 7.07 (t, 1H, *J* = 7.4 and 7.4 Hz, ArH), 7.26 (t, 1H, *J* = 7.2 and 7.5 Hz, ArH), 7.30–7.36 (m, 4H, ArH), 7.60 (d, 1H, *J* = 8.1 Hz, ArH), 7.64 (d, 2H, *J* = 7.6 Hz, ArH), 8.33 (s, 1H, indole), 8.35 (d, 2H, *J* = 7.9 Hz, ArH), 8.77 (s, 1H, −N=CH−), 9.03 (s, 1H, NH), 9.98 (s, 1H, NH), 12.30 (s, H, NH), 12.60 (s, H, NH). ^13^C NMR (DMSO-*d*_6_, 100 MHz, δ ppm): 55.21 (C, OCH_3_), 91.67, 112.92, 114.29, 117.42, 119.30, 121.18, 122.03, 123.19, 123.82, 124.31, 128.93, 137.65, 138.62, 152.83, 155.31, 156.20, 156.60, 158.92 (24C), 163.27 (C, C=O). Anal. calcd. (%) for C_26_H_22_N_6_O_2_ (450.49): C, 69.32; H, 4.92; N, 18.66. Found: C, 69.25; H, 5.00; N, 18.60%.

##### 4.1.2.5 5-((1*H*-Indol-3-yl)methyleneamino)-3-(4-methoxyphenylamino)-*N*-(4-methylphenyl)-1*H*-pyrazole-4-carboxamide (**7e**)

Yellow crystals, mp 258–260 °C, yield (77%). ^1^H NMR (DMSO-*d*_6_, 400 MHz, δ ppm): 2.26 (s, 3H, CH_3_), 3.72 (s, 3H, OCH_3_), 6.89 (d, 2H, *J* = 9.0 Hz, ArH), 7.13 (d, 2H, *J* = 8.3 Hz, ArH), 7.25 (t, 1H, *J* = 7.8 and 7.9 Hz, ArH), 7.34 (t, 1H, *J* = 8.2 and 8.1 Hz, ArH), 7.48 (d, 2H, *J* = 8.5 Hz, ArH), 7.53 (d, 2H, *J* = 8.4 Hz, ArH), 7.59 (d, 1H, *J* = 8.1 Hz, ArH), 8.31 (s, 1H, indole), 8.34 (d, 1H, *J* = 7.7 Hz, ArH), 8.78 (s, 1H, −N=CH−), 9.05 (s, 1H, NH), 9.96 (s, 1H, NH), 12.26 (s, 2H, 2NH). ^13^C NMR (DMSO-*d*_6_, 100 MHz, δ ppm): 20.37 (C, CH_3_), 55.22 (C, OCH_3_), 91.54, 112.90, 114.28, 144.33, 118.23, 119.28, 121.18, 121.94, 123.78, 124.28, 129.30, 132.16, 136.09, 137.52, 137.64, 150.02, 153.20, 153.24, 153.31, 158.37 (24C), 163.12 (C, C=O). MS (*m*/*z*, %): 464 (M^+^, 43.73), 337 (100). Anal. calcd. (%) for C_27_H_24_N_6_O_2_ (464.52): C, 69.81; H, 5.21; N, 18.09. Found: C, 69.75; H, 5.25; N, 18.00%.

### 4.2 Biological Evaluation

#### 4.2.1 *In Vitro* Anticancer Activity

##### 4.2.1.1 Cell Culture Conditions

The cells of human liver carcinoma (HepG2), human breast adenocarcinoma (MCF-7), human colorectal carcinoma (HCT-116), and human lung carcinoma (A549) were purchased from the American Type Culture Collection (Rockville, MD). All cells were maintained in a Dulbecco’s modified Eagle’s medium (DMEM), which was supplemented with 10% of heat-inactivated fetal bovine serum (FBS) and 100 U/mL penicillin and streptomycin each. The cells were grown at 37 °C in a humidified atmosphere of 5% CO_2_.

##### 4.2.1.2 MTT Cytotoxicity Assay

The cytotoxicity activities on the human liver carcinoma (HepG2), human breast adenocarcinoma (MCF-7), human colorectal carcinoma (HCT-116), and human lung carcinoma (A549) cell lines were estimated employing the 3-(4,5-dimethyl-2-thiazolyl)-2,5-diphenyl-2*H*-tetrazolium bromide (MTT) assay, which was grounded on the reduction of the tetrazolium salt by mitochondrial dehydrogenases in viable cells.^51,52^ The cells were dispensed in a 96-well sterile microplate (3 × 10^4^ cells/well), followed by their incubation at 37 °C with a series of different concentrations of 10 μL of each compound or doxorubicin (positive control, in DMSO) for 48 h in a serum-free medium prior to the MTT assay. Subsequently, the media were carefully removed, and 40 μL of MTT (2.5 mg/mL) was added to each well and then incubated for an additional 4 h. Purple formazan dye crystals were solubilized by the addition of 200 μL of DMSO. The absorbance was measured at 570 nm using a SpectraMax Paradigm Multi-Mode microplate reader. The relative cell viability was expressed as the mean percentage of viable cells relative to the untreated control cells. All experiments were conducted in triplicate and were repeated on three different days. All of the values were represented as mean ± standard deviation (SD). The IC_50_s were determined by the SPSS probit analysis software program (SPSS Inc., Chicago, IL).

#### 4.2.2 Cell Cycle Analysis and Apoptosis Detection

Cell cycle analysis and apoptosis detection were carried out using flow cytometry.^62^ Both HepG2 and MCF-7 cells were seeded at 8 × 10^4^ and incubated at 37 °C and 5% CO_2_ overnight. After treatment with the tested compounds for 24 h, cell pellets were collected and centrifuged (300*g*, 5 min). For cell cycle analysis, the cell pellets were fixed with 70% ethanol on ice for 15 min and collected again. The collected pellets were incubated with propidium iodide (PI) staining solution (50 μg/mL PI, 0.1 mg/mL RNaseA, 0.05% Triton X-100) at room temperature for 1 h and analyzed using a Gallios flow cytometer (Beckman Coulter, Brea, CA). Apoptosis detection was performed using a FITC annexin V/PI commercial kit (Becton Dickenson, Franklin Lakes, NJ) following the manufacturer’s protocol. The samples were analyzed using fluorescence-activated cell sorting (FACS) with a Gallios flow cytometer (Beckman Coulter, Brea, CA) within 1 h after staining. Data were analyzed using Kaluza v1.2 (Beckman Coulter). All monolayers of cells were treated separately for 48 h with DMSO or the IC_50_ of compounds **7a** and **7b**.

#### 4.2.3 Enzymatic Assay

##### 4.2.3.1 Caspase-3 Assay

Activities of caspase-3 were measured using the Invitrogen caspase-3 (Active) (human) ELISA kit, Catalog # KHO1091 (96 tests) (Invitrogen Corporation) according to the manufacturer’s instructions.

##### 4.2.3.2 Bcl-2 ELISA Assay

Activities of Bcl-2 were measured using the Invitrogen Zymed Bcl-2 ELISA Kit, Catalog # 99–0042 (96 tests) (Invitrogen Corporation) according to the manufacturer’s instructions.

##### 4.2.3.3 Bax ELISA Assay

Activities of Bax were measured using the Human Bax ELISA kit (DRG Human Bax ELISA (EIA-4487) DRG International, Inc.) according to the manufacturer’s instructions.

##### 4.2.3.4 *In Vitro* CDK-2 Enzyme Inhibitory Assessment

Estimation of CDK-2 was performed using ELISA through an affinity tag labeled capture antibody and a reporter conjugated detector antibody, which immunocapture the sample analyte in solution. The addition of the standard and samples to the wells is carried out, followed by the addition of the antibody mix. After the incubation period is completed, the wells are washed, and the unrestrained substance is discarded. Then, TMB (3,3′,5,5′-tetramethylbenzidine) substrate is added, and prompted by horseradish peroxidase (HRP), blue coloration appeared. The reaction was stopped by the addition of a stop solution, completely changing the color from blue to yellow. Signals were created equivalently to the quantity of the bound analyte, and the intensity was recorded at a certain wavelength (450 nm) using a Robonik P2000 ELISA reader. The concentrations of the tested compounds were calculated from the plotted curve.

### 4.3 Molecular Docking Study

Molecular docking studies were carried out using Molecular Operating Environment (MOE, 10.2008) software. The X-ray crystal structure of CDK-2 (PDB code: 2A4L)^61^ was complexed with roscovitine, which was retrieved from the RCSB Protein Data Bank. All structure minimizations were performed with MOE until an RMSD gradient of 0.05 kcal/(mol Å) with an MMFF94x force field and the partial charges were automatically calculated. The structure of the CDK-2 enzyme was prepared for molecular docking using Protonate 3D protocol in MOE with the default options. The Triangle Matcher placement method and the London dG scoring function were applied in the docking protocol. First, the validation process was confirmed by redocking the native ligand, followed by docking of the compounds **7a** and **7b** into the active site after removing the co-crystallized ligand following the reported procedure.^63^

## Supporting Information Available

^1^H and ^13^C NMR spectra of the synthesized pyrazole–indole hybrids (PDF)
