# Supplementary material for: Design, Synthesis, Anticancer Evaluation, Enzymatic Assays, and a Molecular Modeling Study of Novel Pyrazole–Indole Hybrids
Source: ACS Omega. 2021 Apr 29;6(18):12361–74. doi: 10.1021/acsomega.1c01604 (PMC8154124; doi:10.1021/acsomega.1c01604)
Supplement: Supplementary file 1 — ao1c01604_si_001.pdf [file ao1c01604_si_001.pdf]

SUPPLEMENTARY MATERIAL

Design, Synthesis, Anticancer Evaluation, Enzymatic Assays, and a Molecular Modeling Study of Novel Pyrazole-Indole Hybrids

Ashraf S. Hassan[1], Gaber O. Moustafa[2], Hanem M. Awad[3], Eman S. Nossier[4], Mohamed F. Mady*[5,6]

Authors:

[1]*Organometallic and Organometalloid Chemistry Department, National Research Centre, Dokki 12622, Cairo, Egypt.*

[2]*Peptide Chemistry Department, National Research Centre, Dokki 12622, Cairo, Egypt .*

[3]*Department of Tanning Materials and Leather Technology, National Research Centre, Dokki 12622, Cairo, Egypt.*

[4]*Department of Pharmaceutical Chemistry, Faculty of Pharmacy (Girls), Al-Azhar University, 11754 Cairo, Egypt.*

[5]*Department of Chemistry, Bioscience and Environmental Engineering, Faculty of Science and Technology, University of Stavanger, N-4036 Stavanger, Norway.*

[6]*Green Chemistry Department, National Research Centre, Dokki 12622, Cairo, Egypt.*

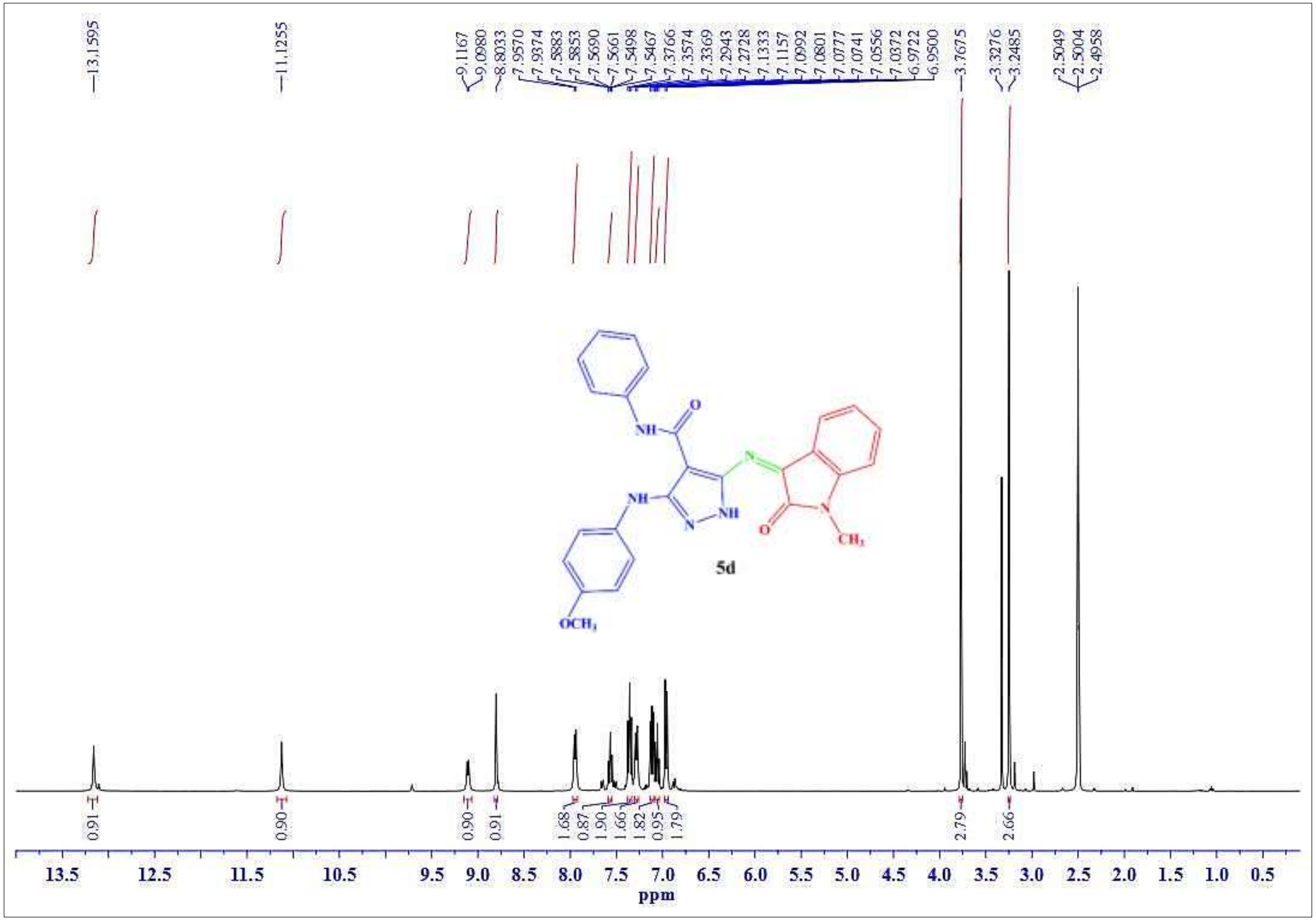

Figure S1: ^{1}H NMR Spectrum of compound **5d**

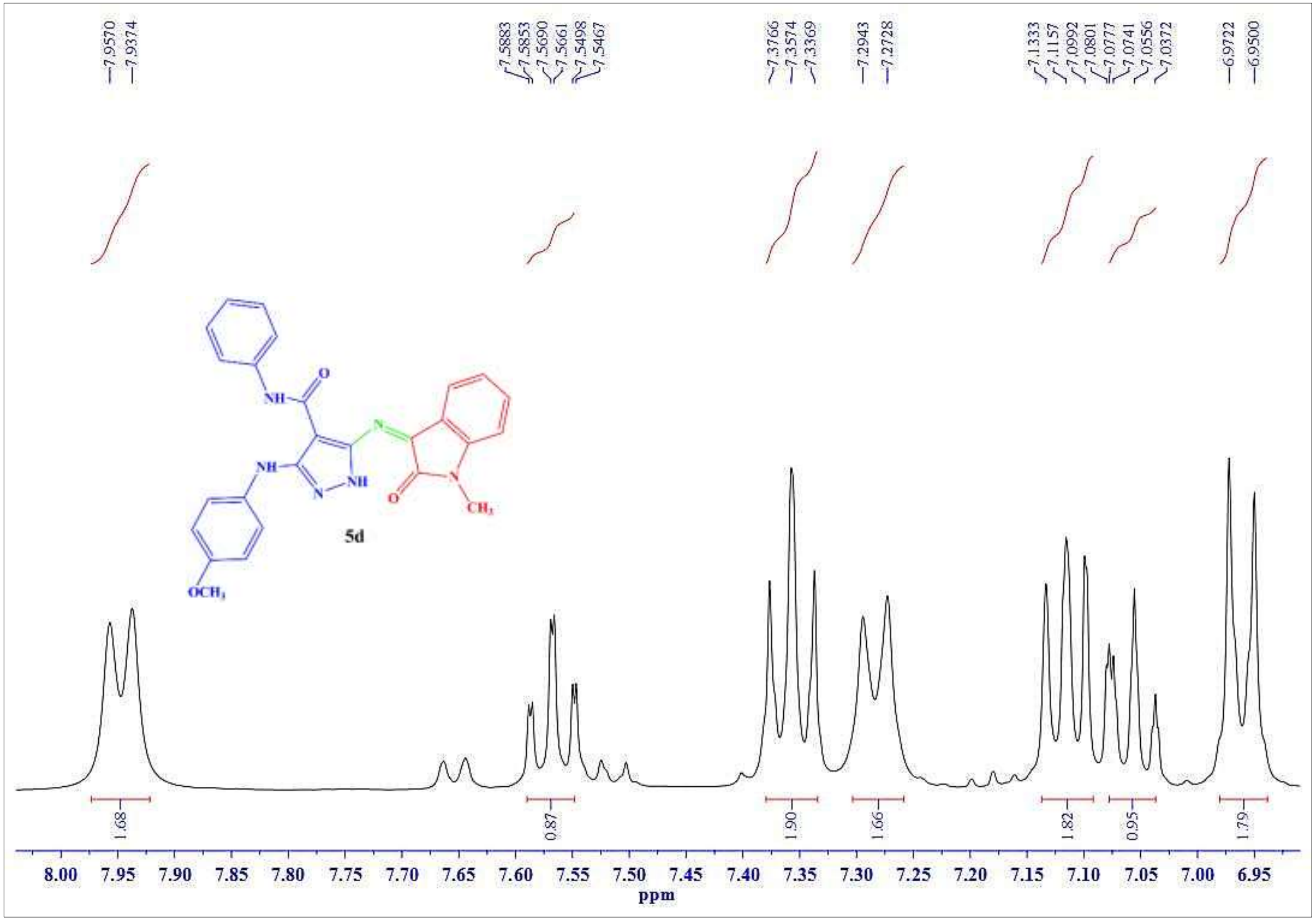

Figure S2: ^{1}H NMR Aromatic region spectrum of compound **5d**

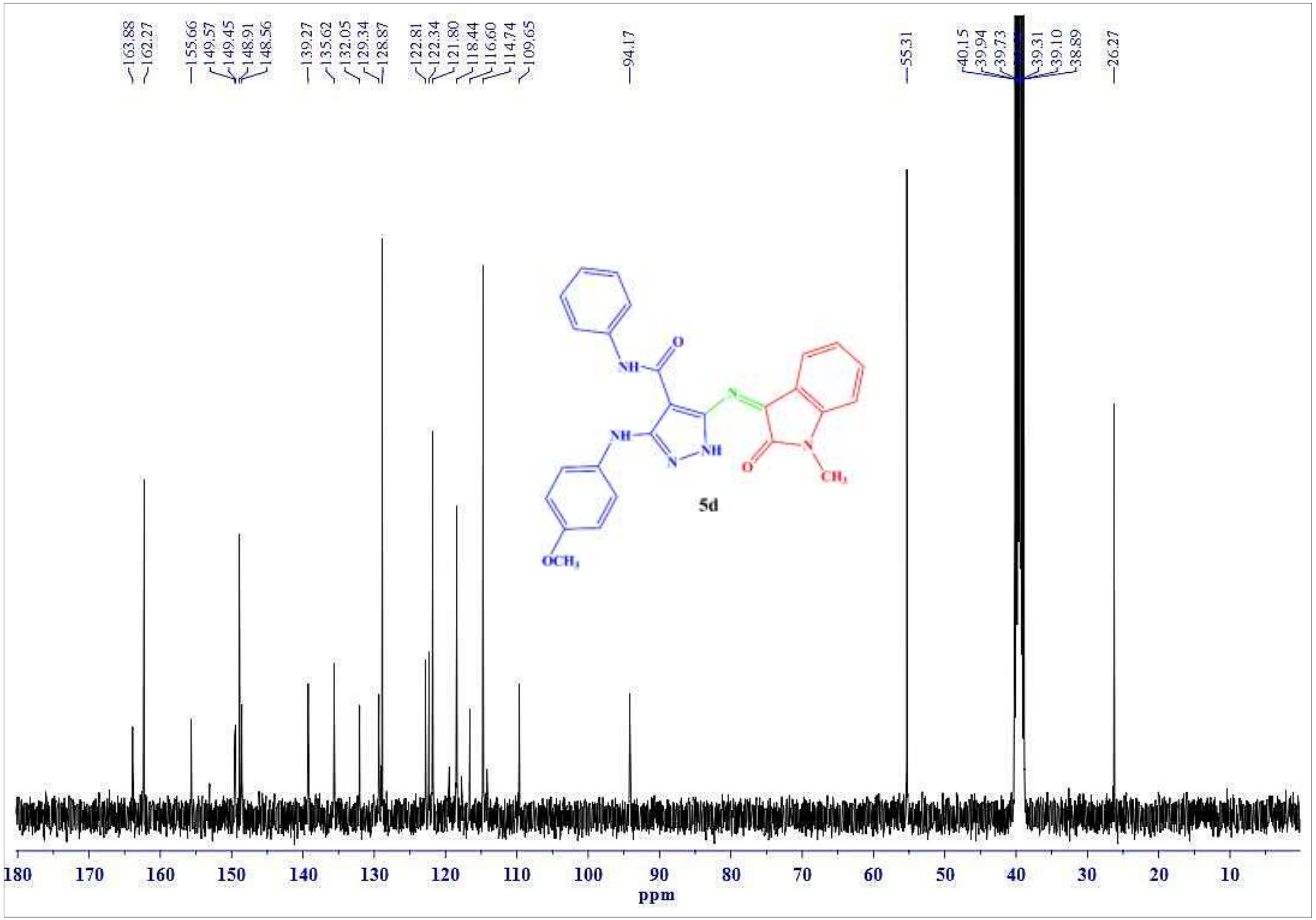

Figure S3: ^{13}C NMR Spectrum of compound **5d**

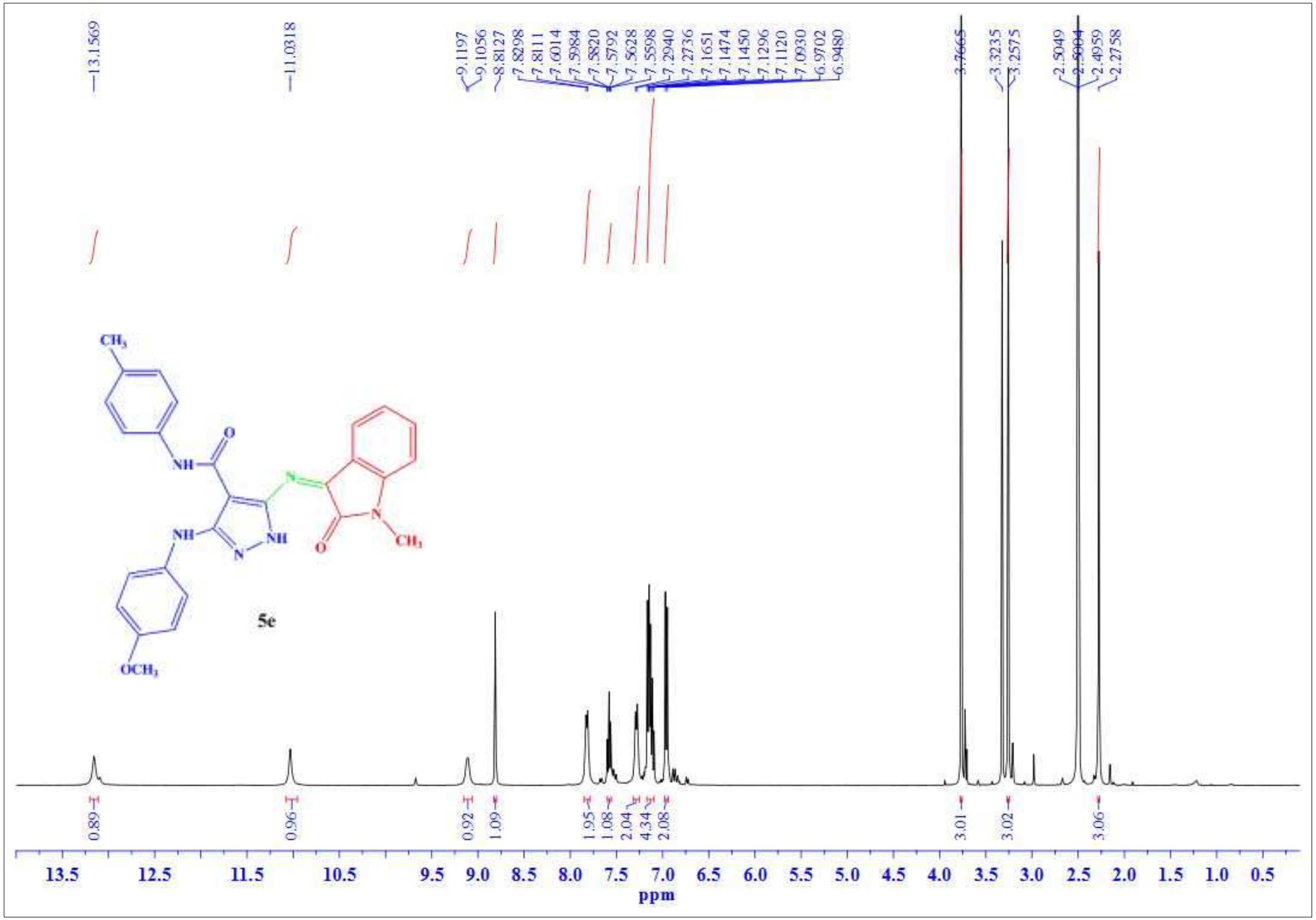

Figure S4: ^{1}H NMR Spectrum of compound **5e**

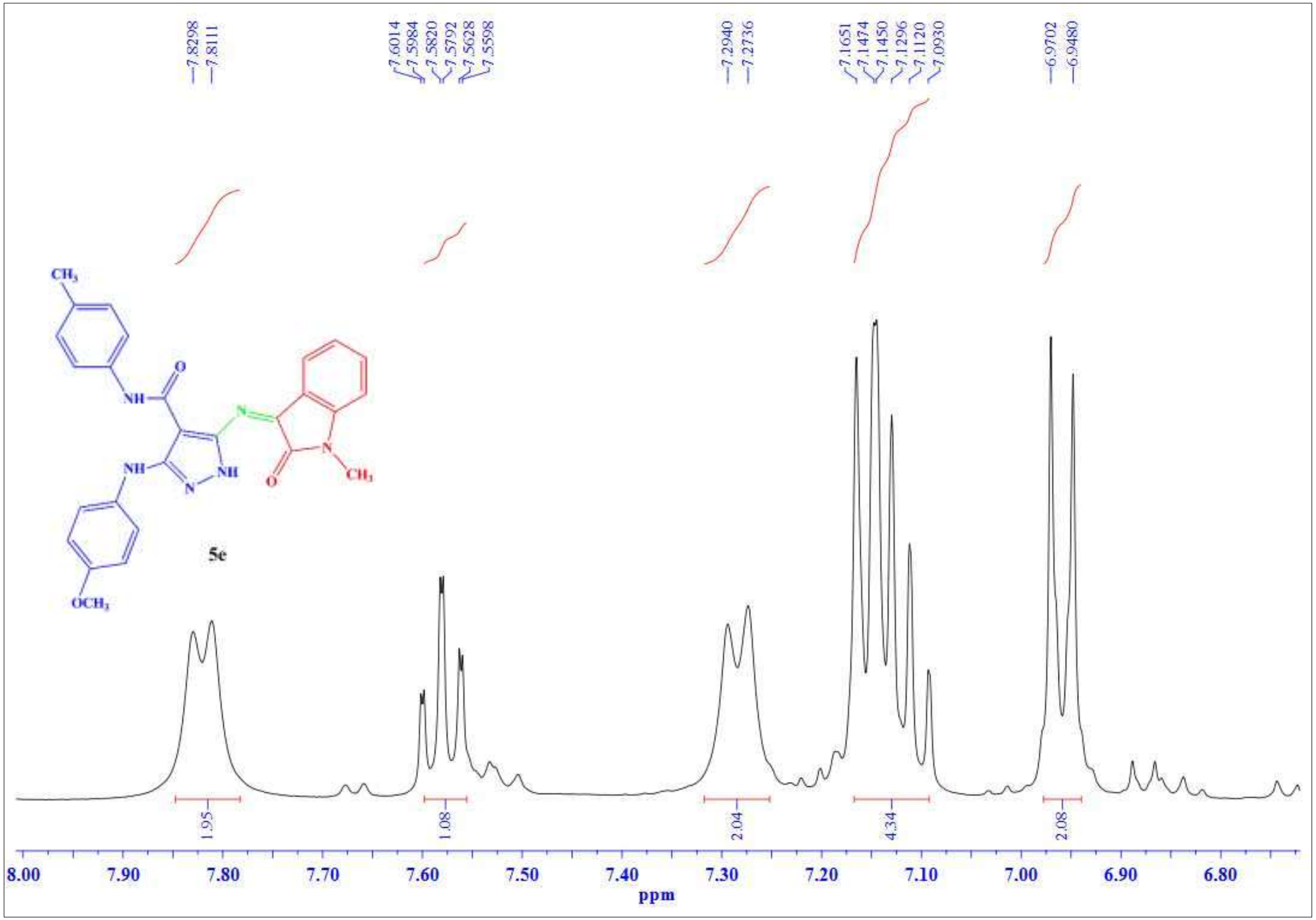

Figure S5: ^{1}H NMR Aromatic region spectrum of compound **5e**

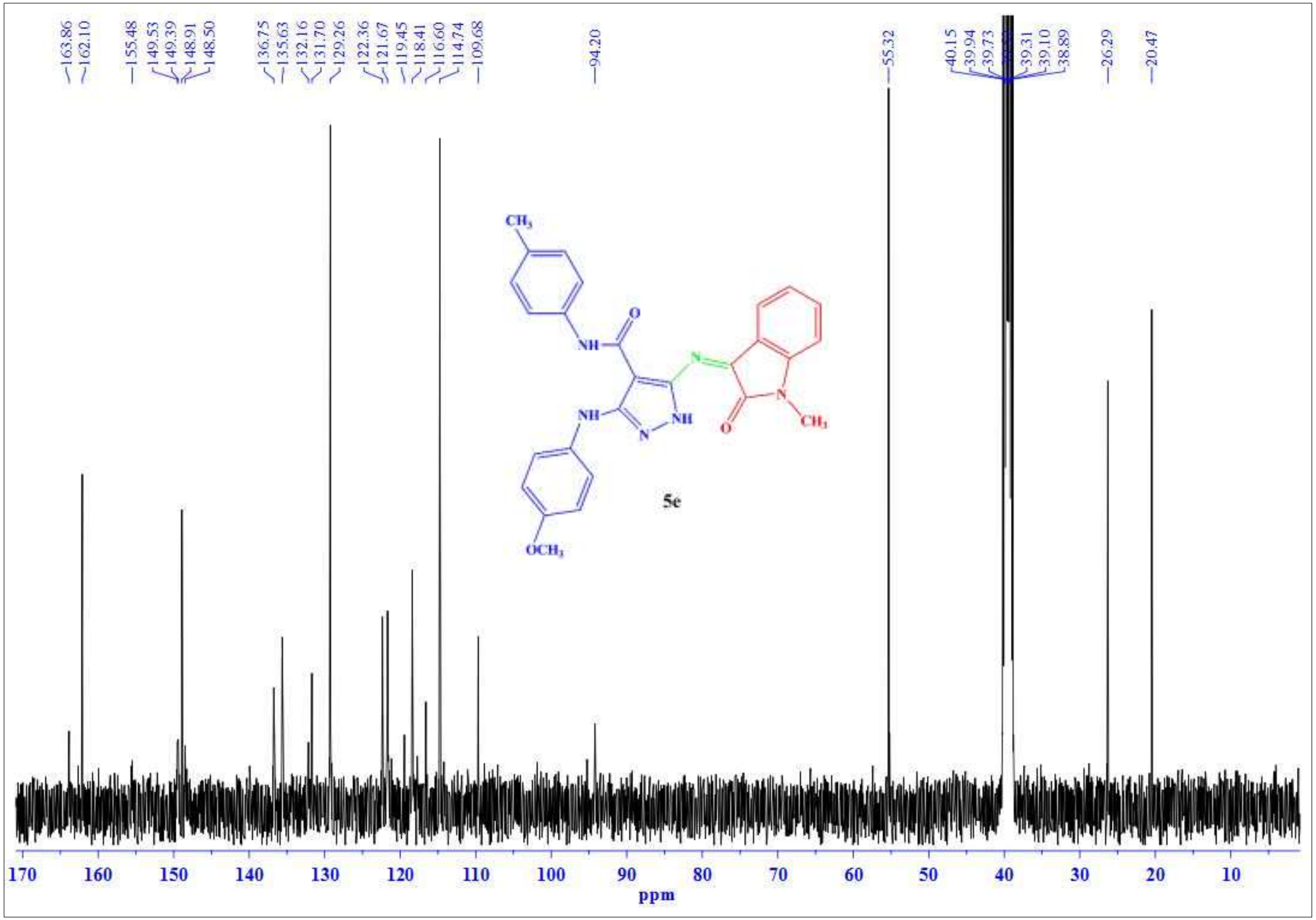

Figure S6: ^{13}C NMR Spectrum of compound **5e**

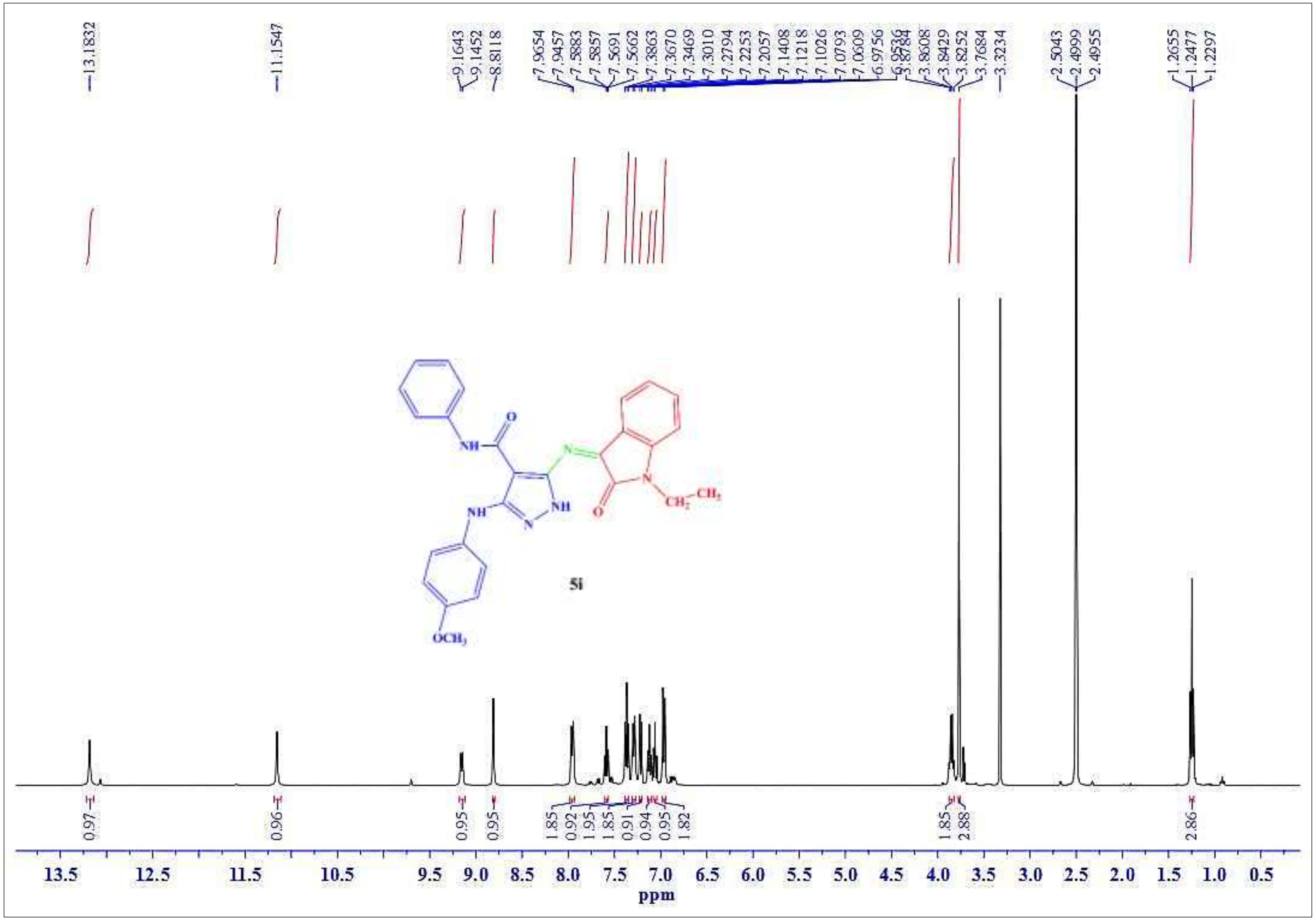

Figure S7: ^{1}H NMR Spectrum of compound **5i**

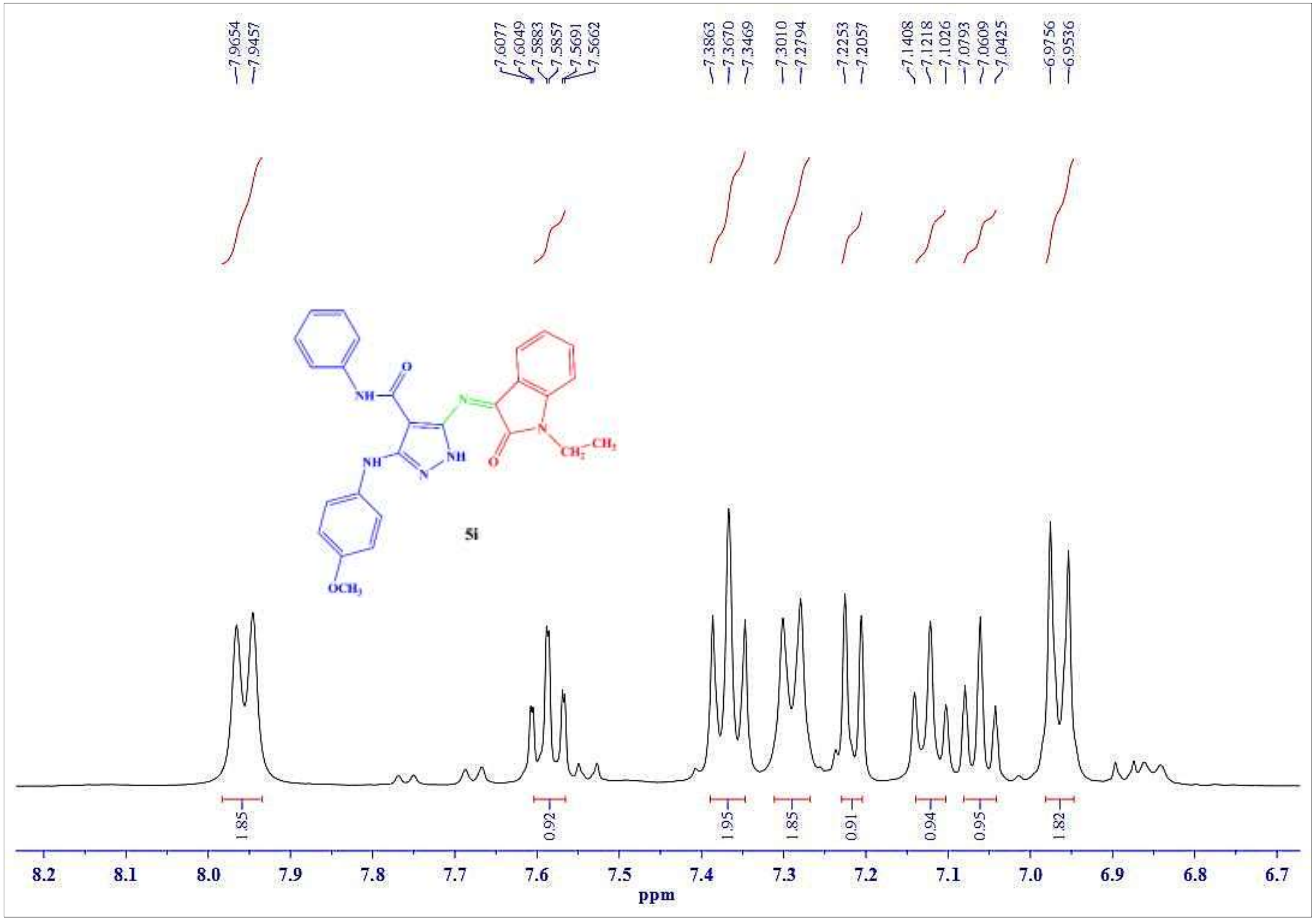

Figure S8: ^{1}H NMR Aromatic region spectrum of compound **5i**

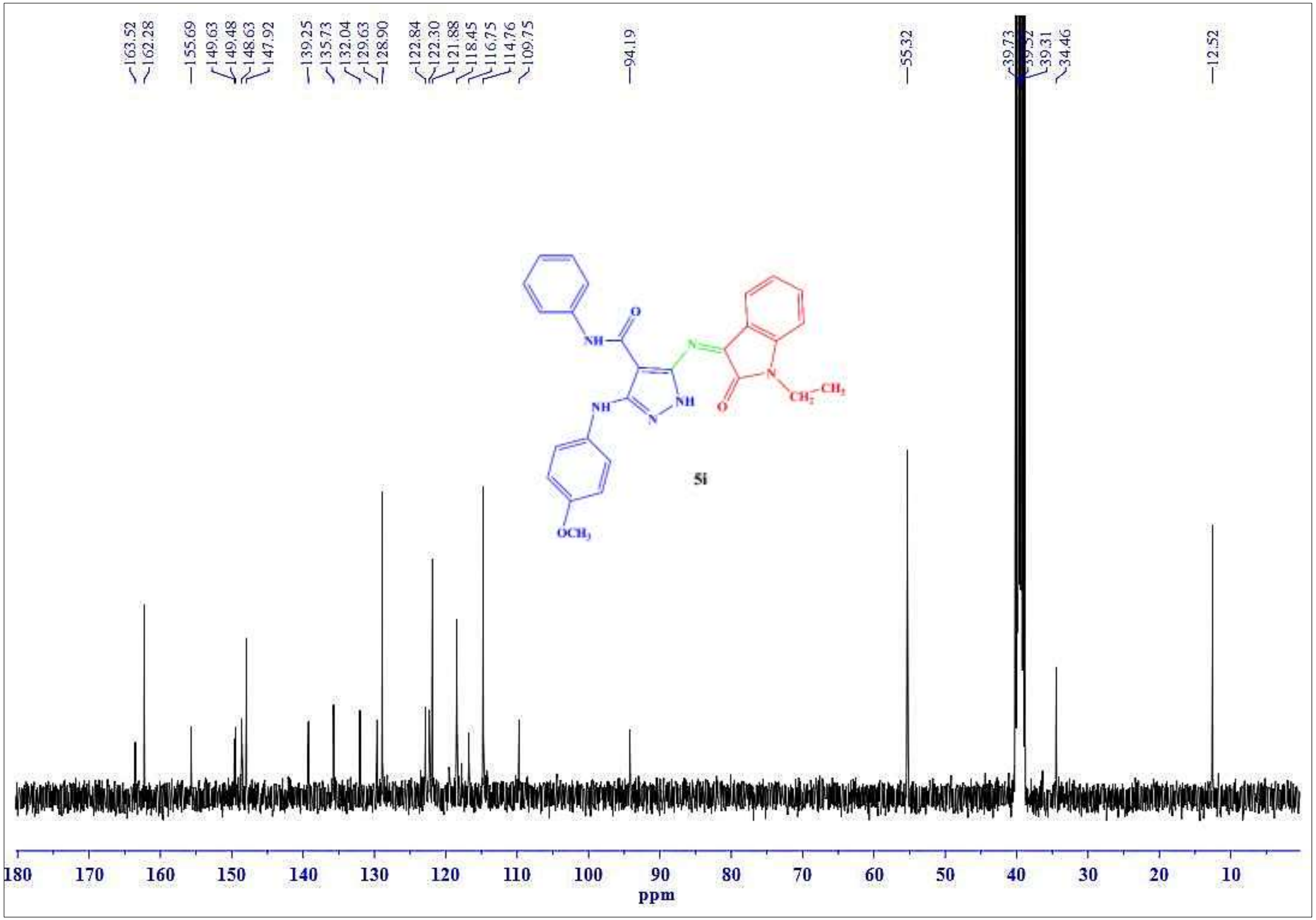

Figure S9: ^{13}C NMR Spectrum of compound **5i**

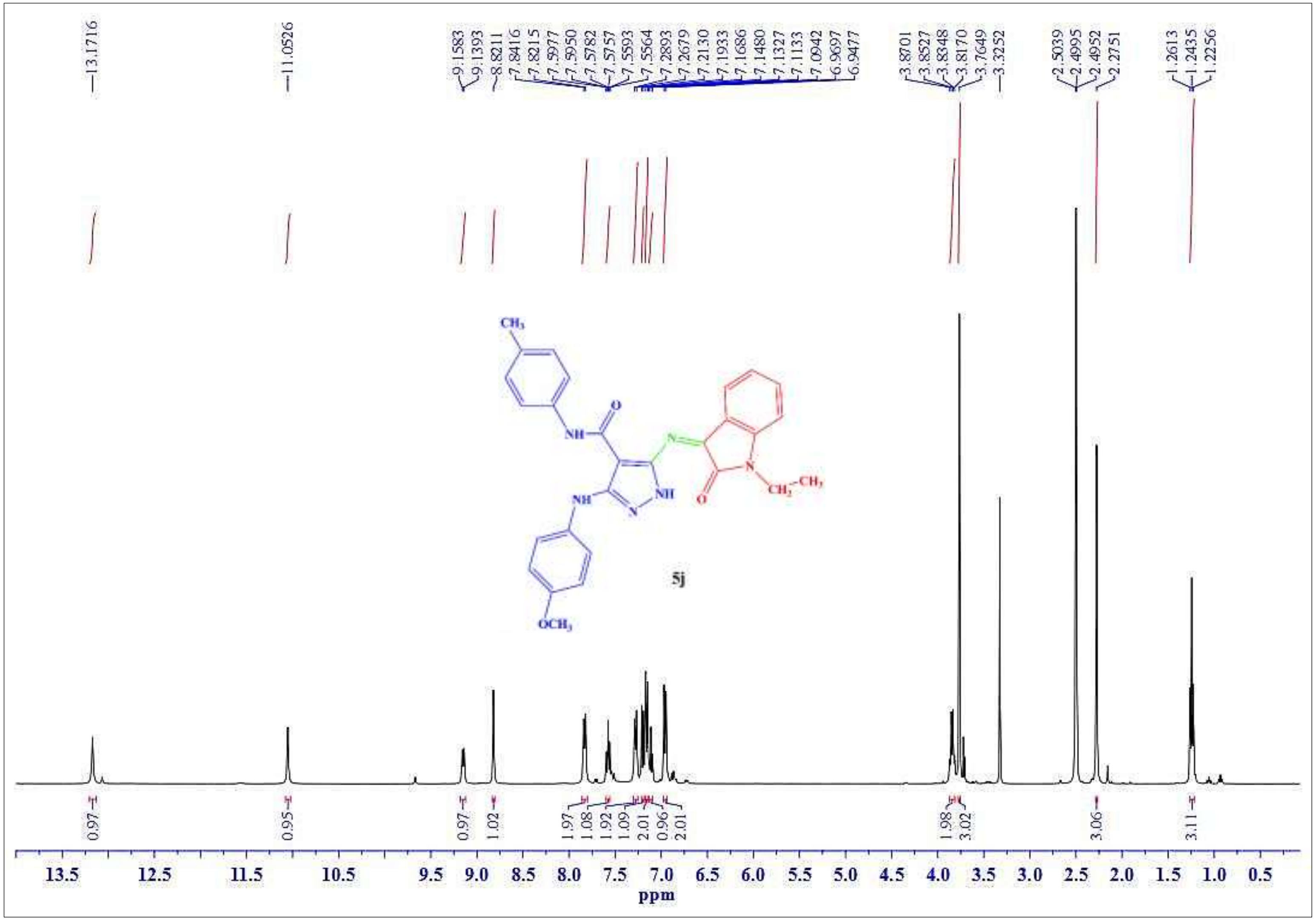

Figure S10: ^{1}H NMR Spectrum of compound **5j**

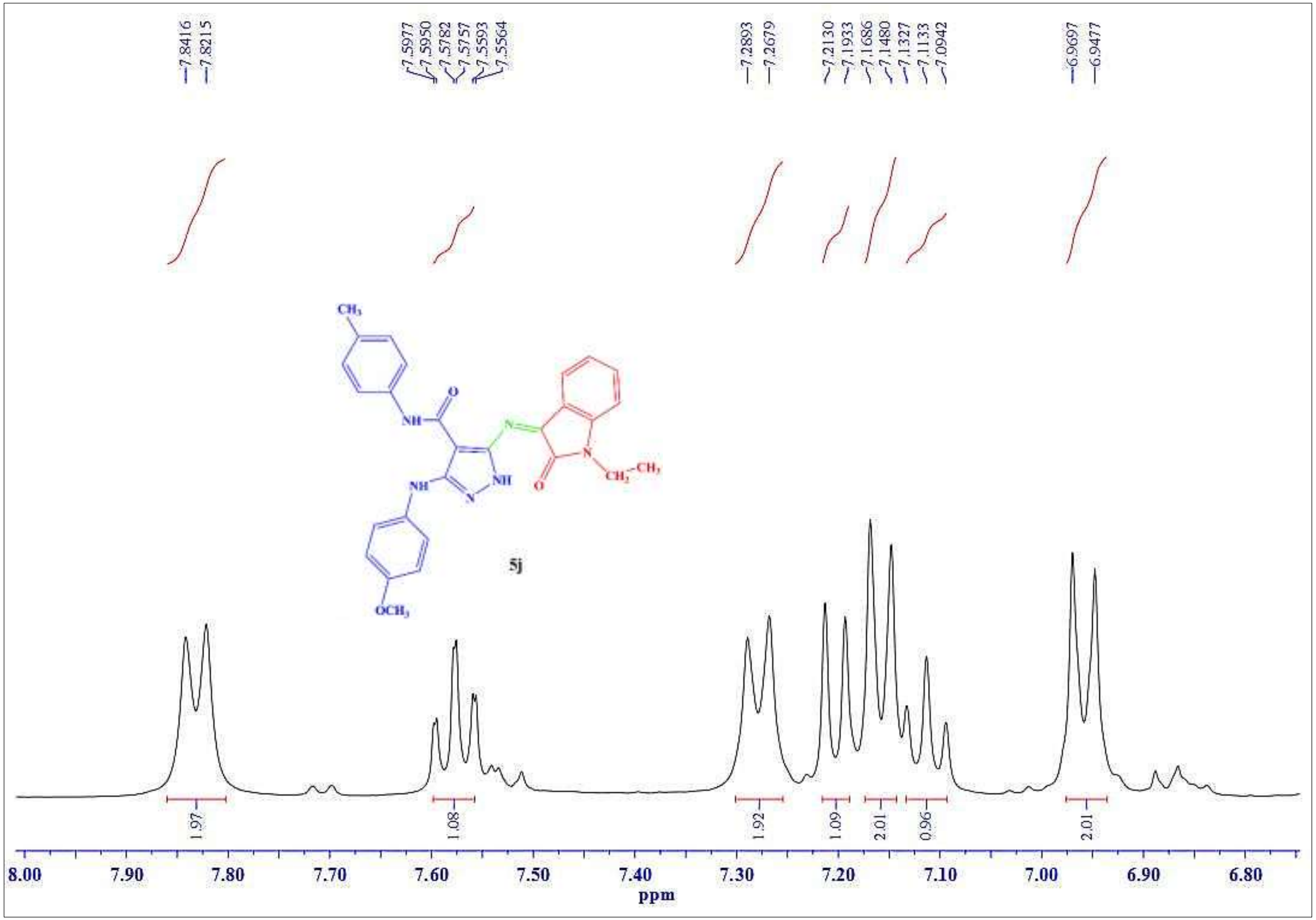

Figure S11: ^{1}H NMR Aromatic region spectrum of compound **5j**

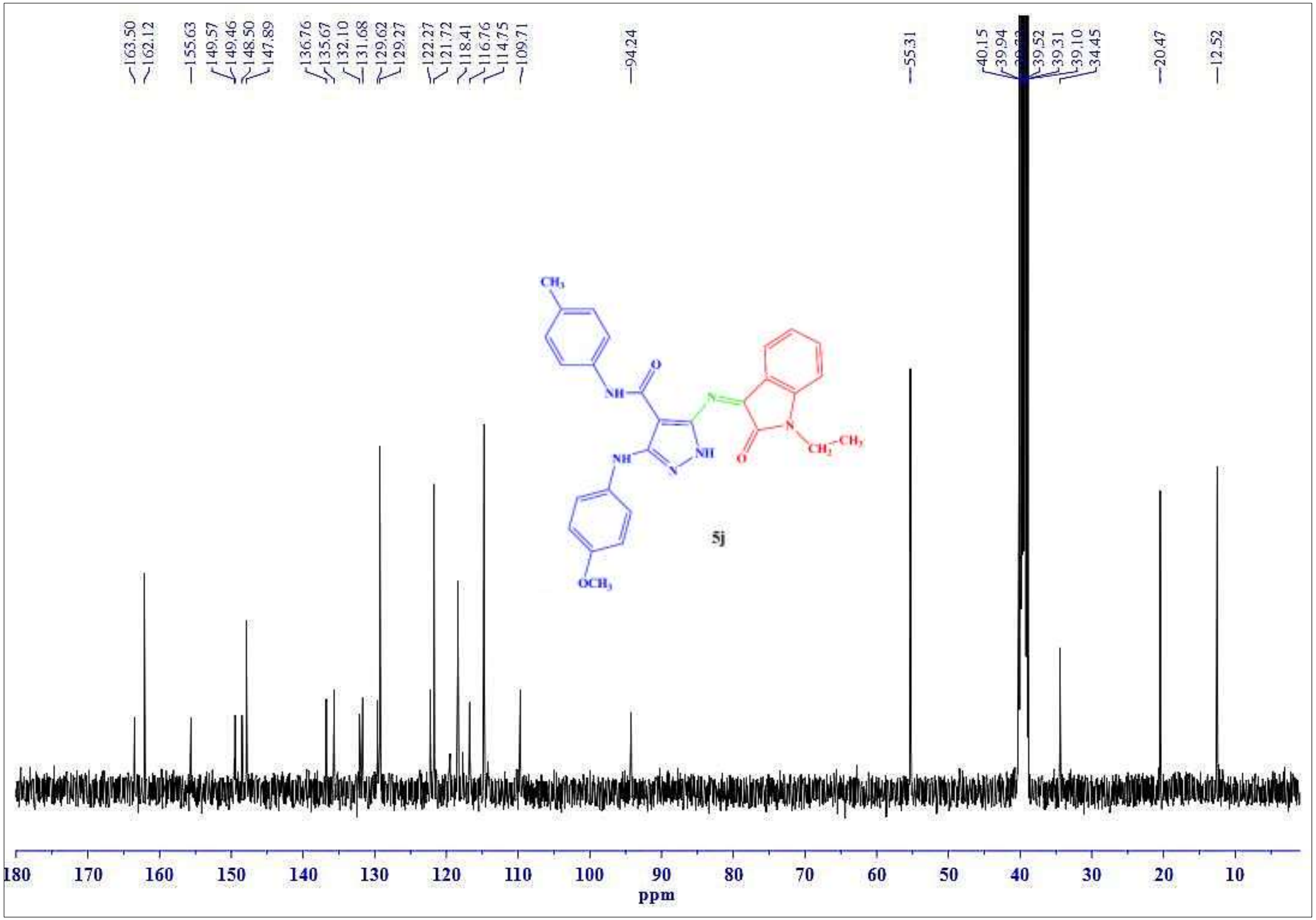

Figure S12: ^{13}C NMR Spectrum of compound **5j**

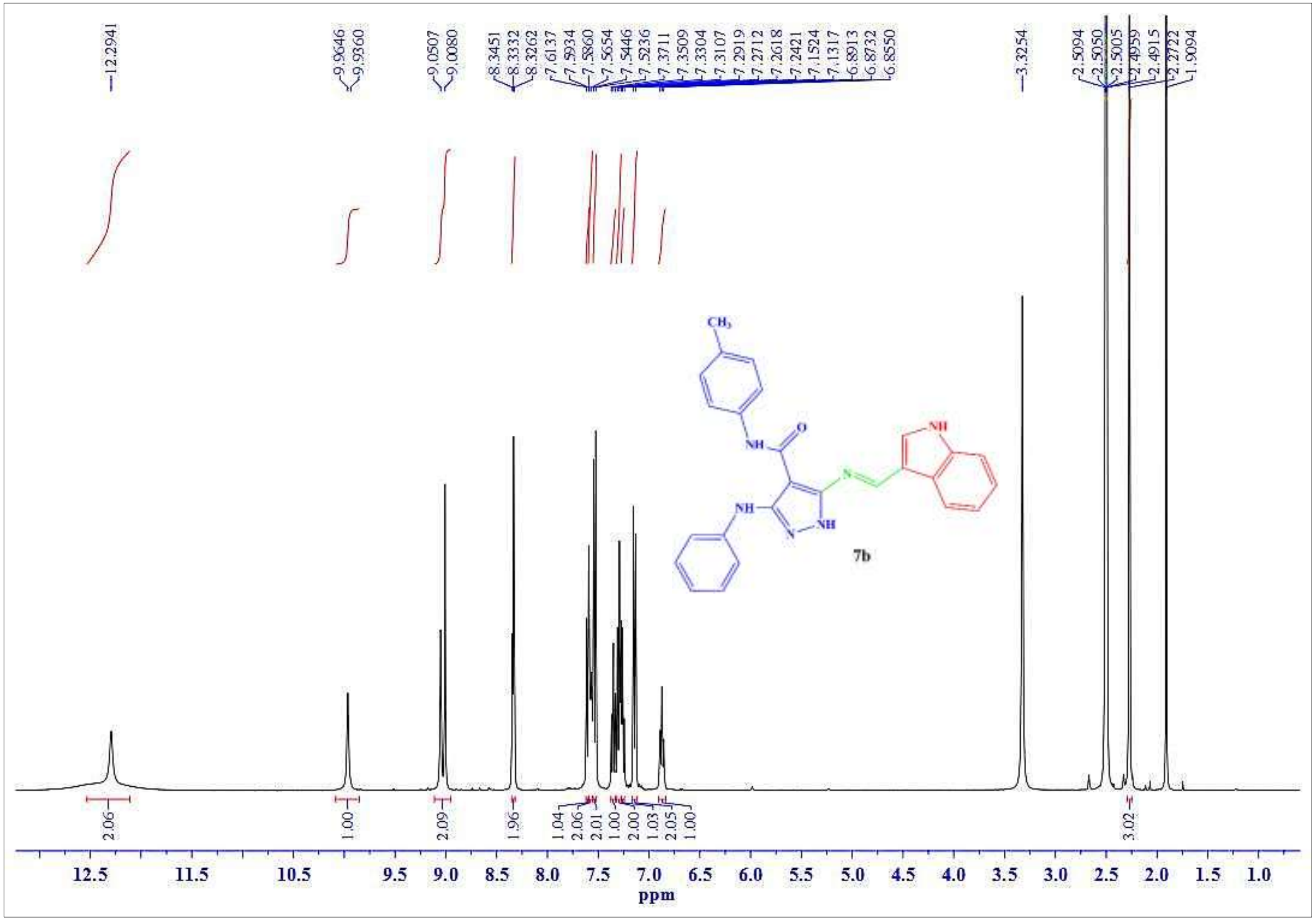

Figure S13: ^{1}H NMR Spectrum of compound **7b**

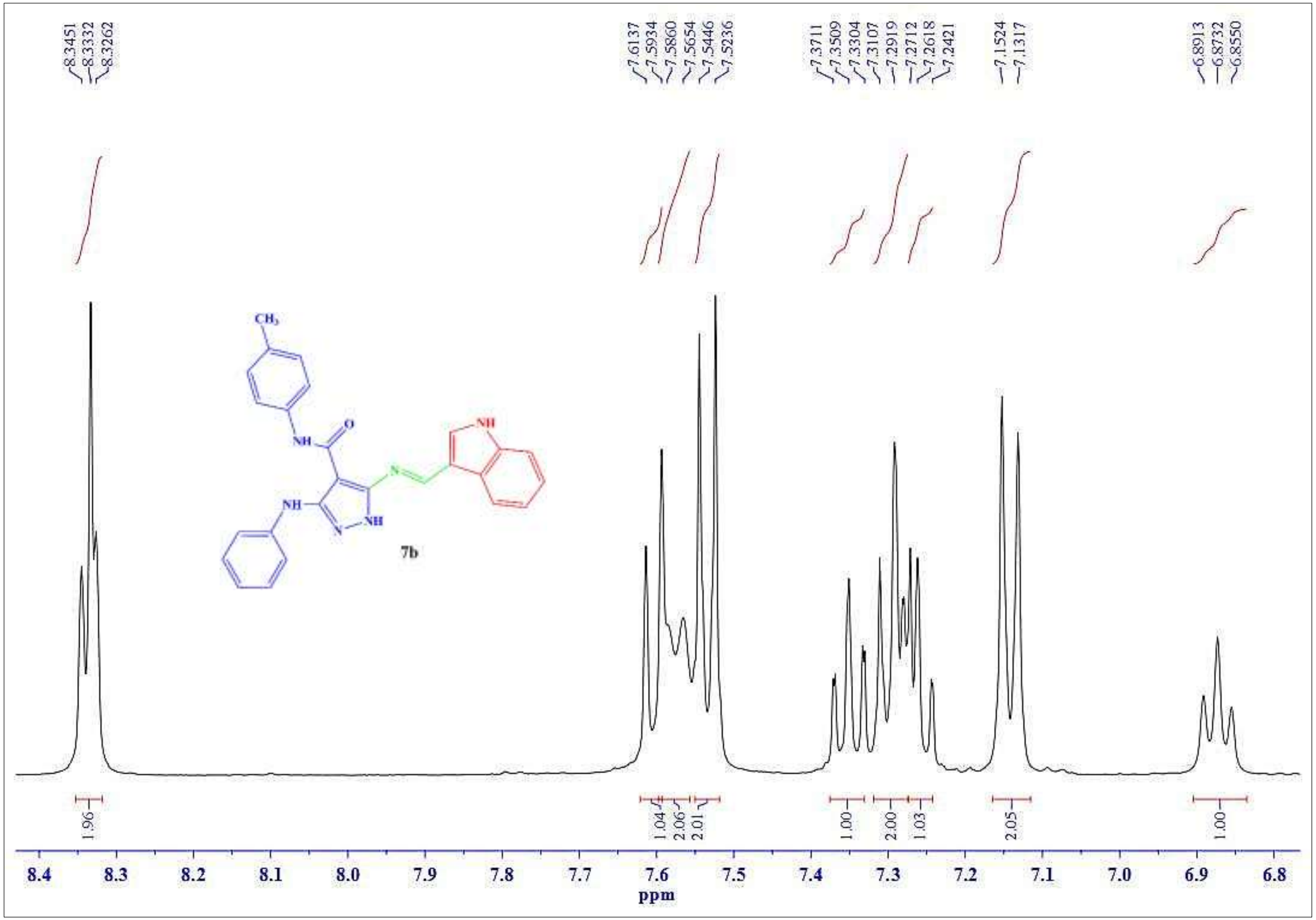

Figure S14: ^{1}H NMR Aromatic region spectrum of compound **7b**

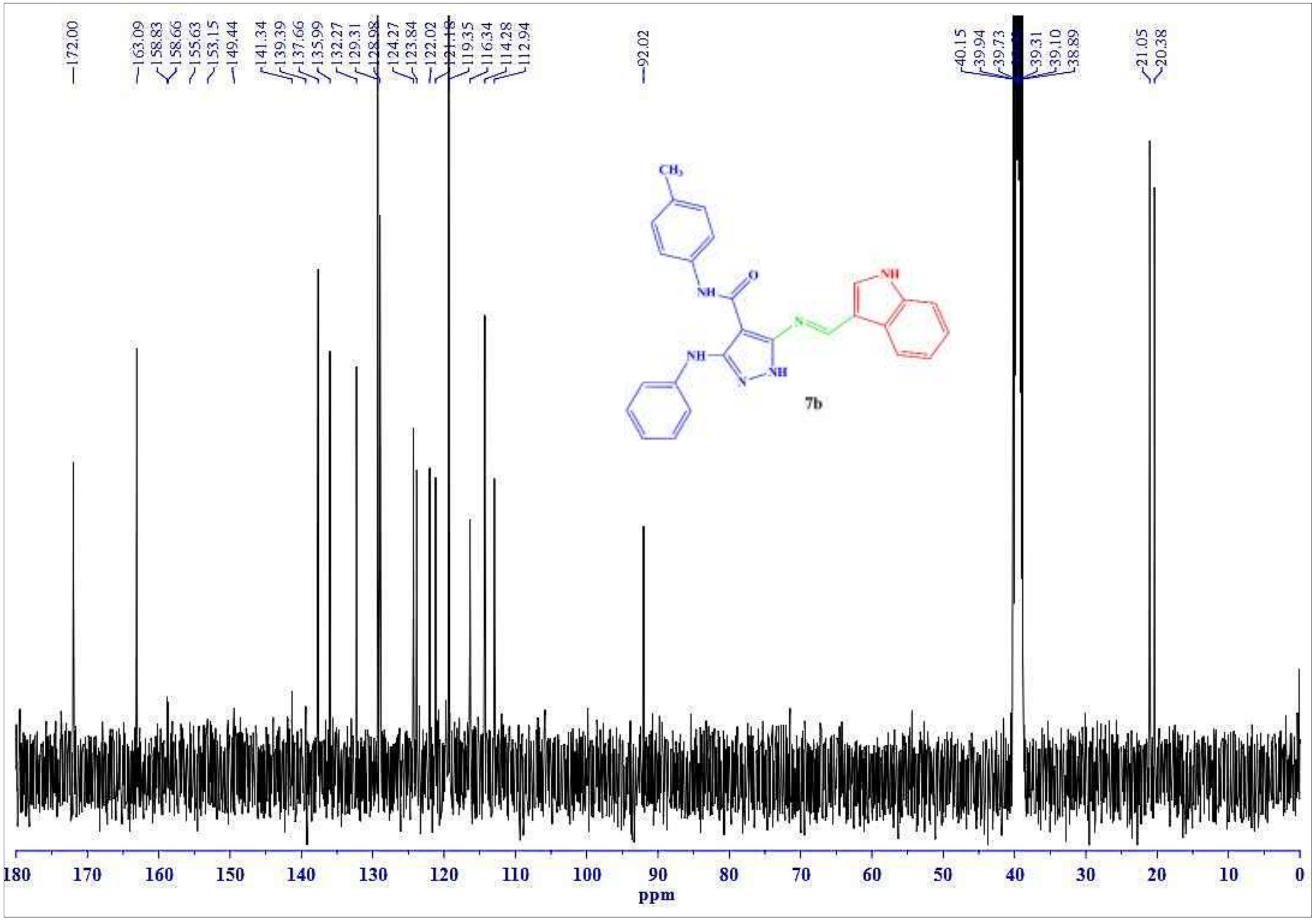

Figure S15: ^{13}C NMR Spectrum of compound **7b**

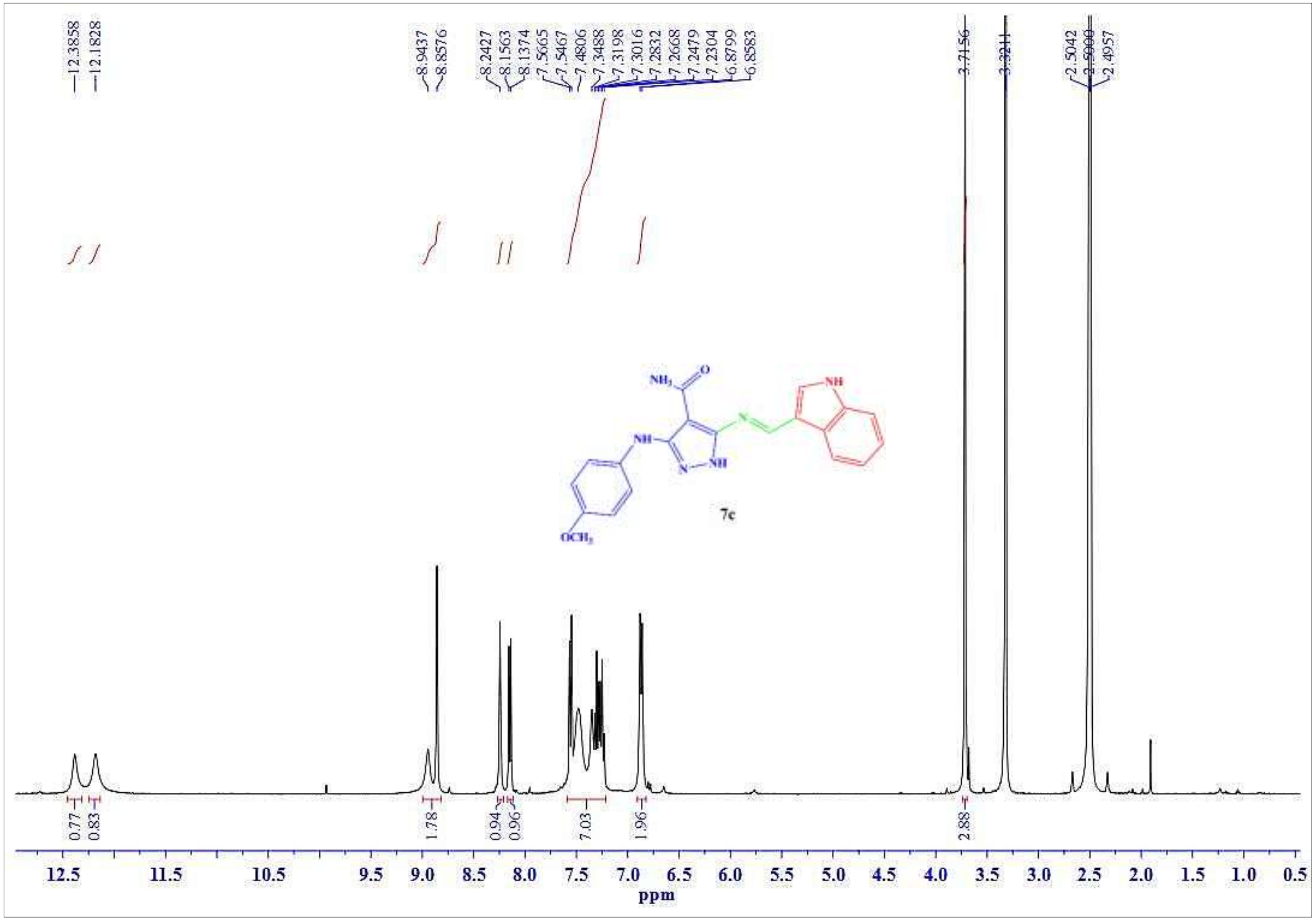

Figure S16: ^{1}H NMR Spectrum of compound **7c**

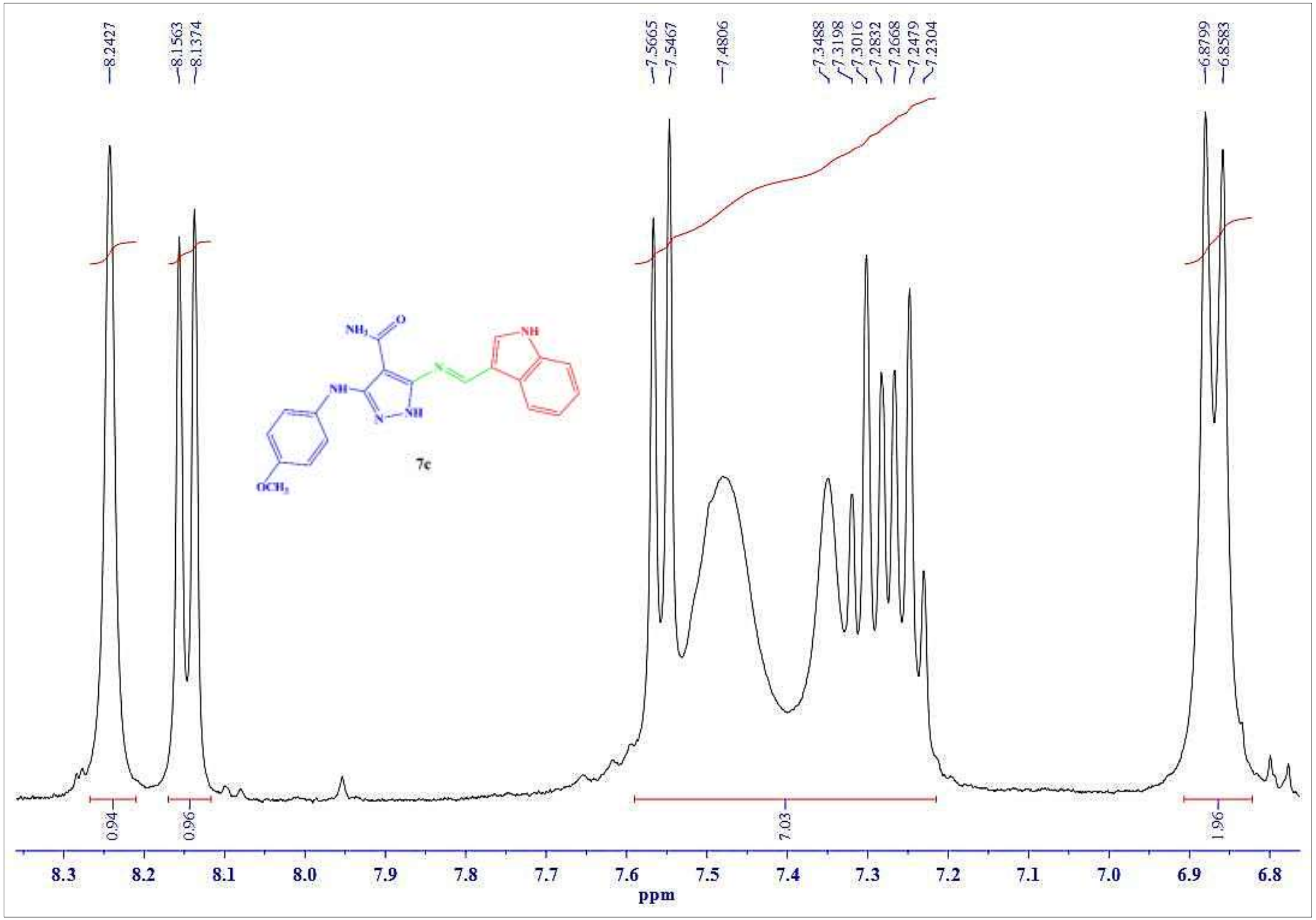

Figure S17: ^{1}H NMR Aromatic region spectrum of compound **7c**

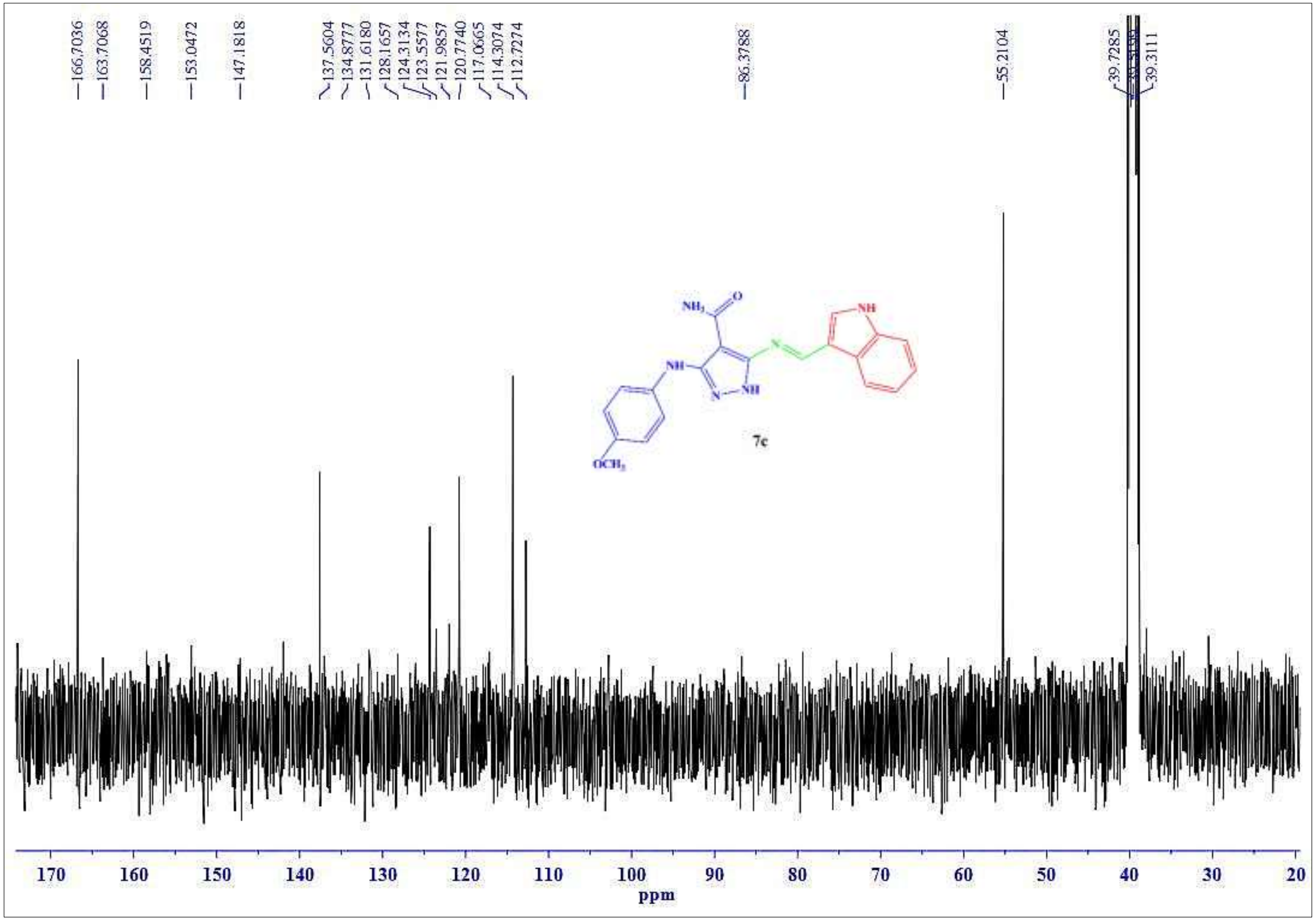

Figure S18: ^{13}C NMR Spectrum of compound **7c**

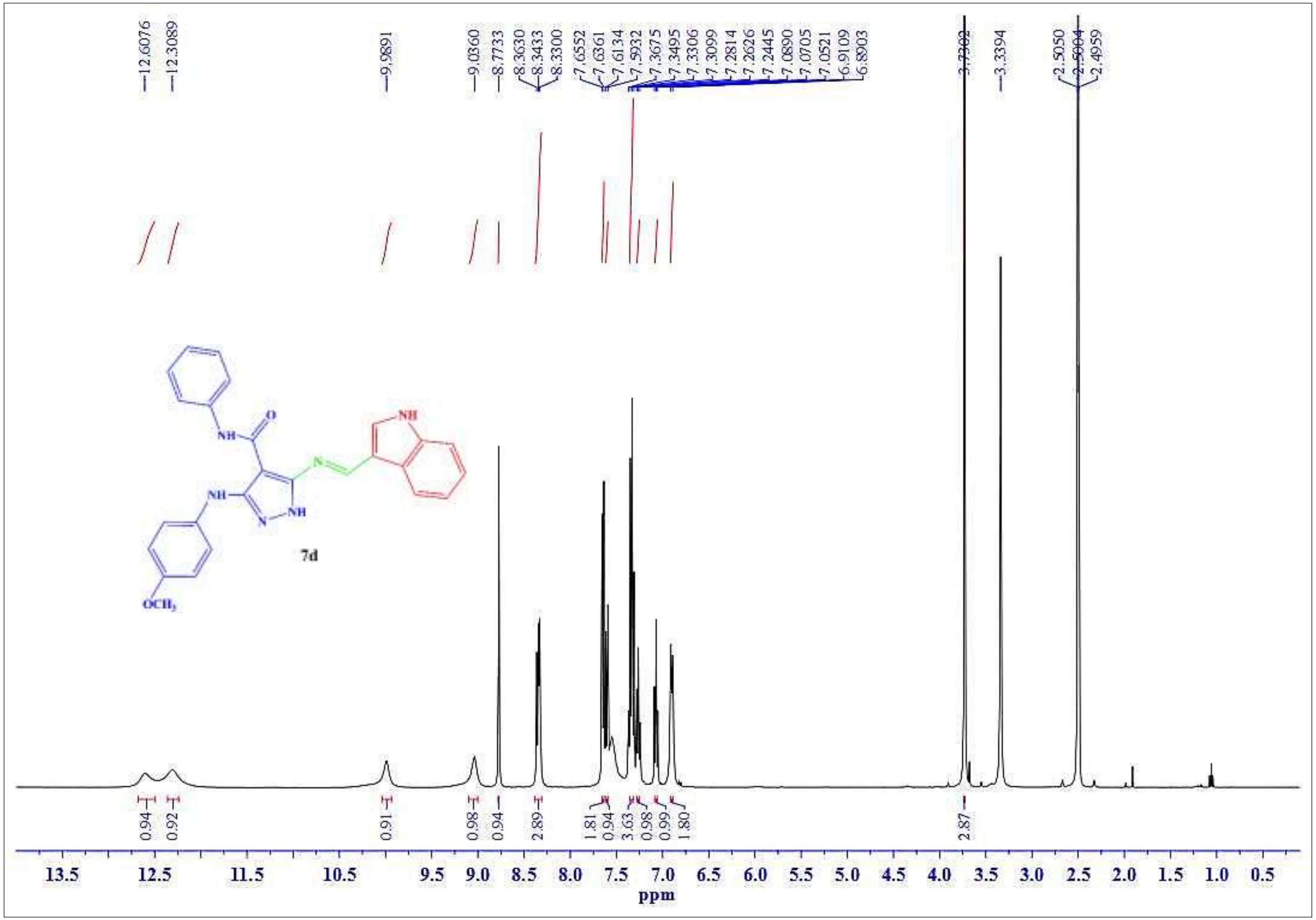

Figure S19: ^{1}H NMR Spectrum of compound **7d**

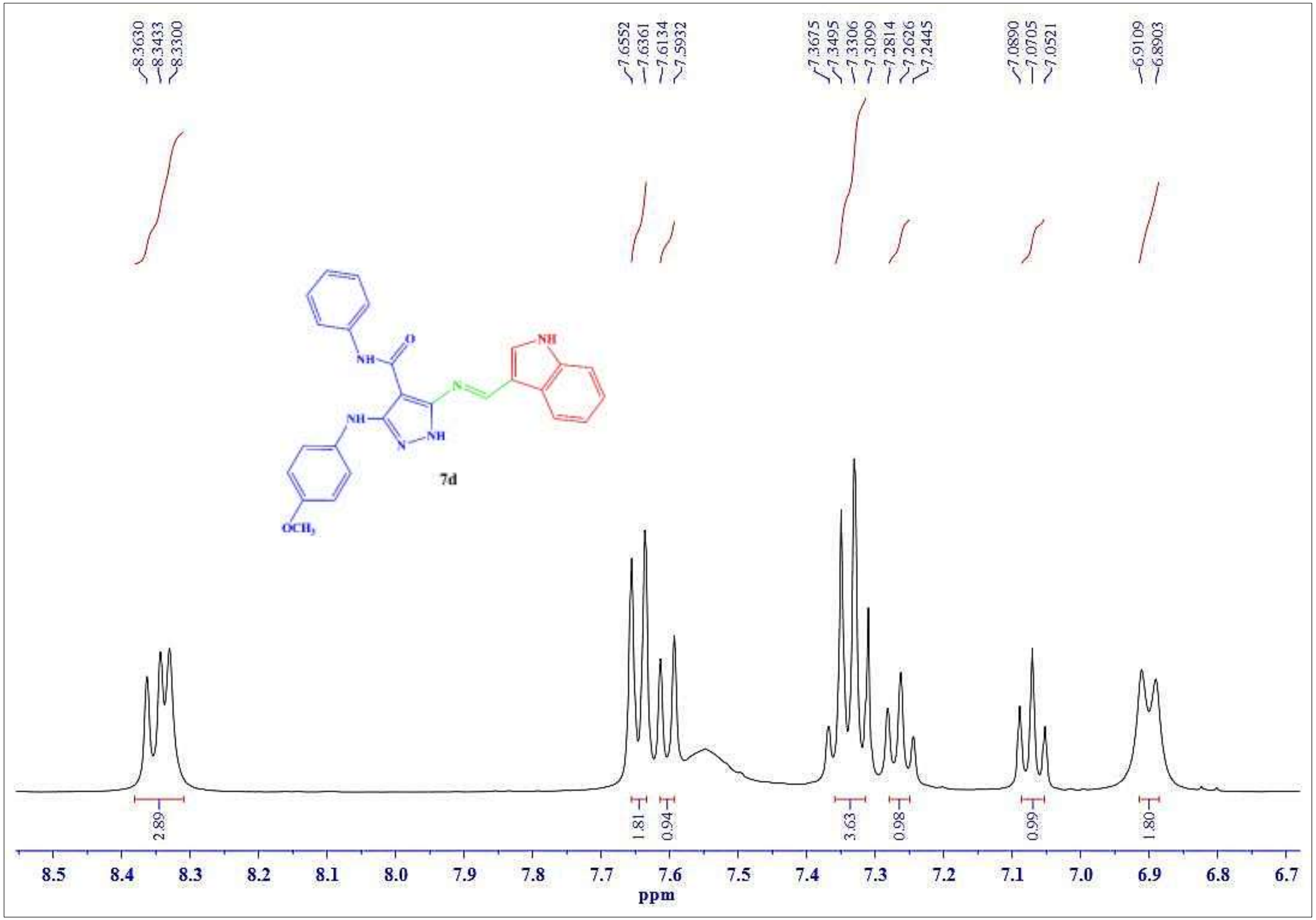

Figure S20: ^{1}H NMR Aromatic region spectrum of compound **7d**

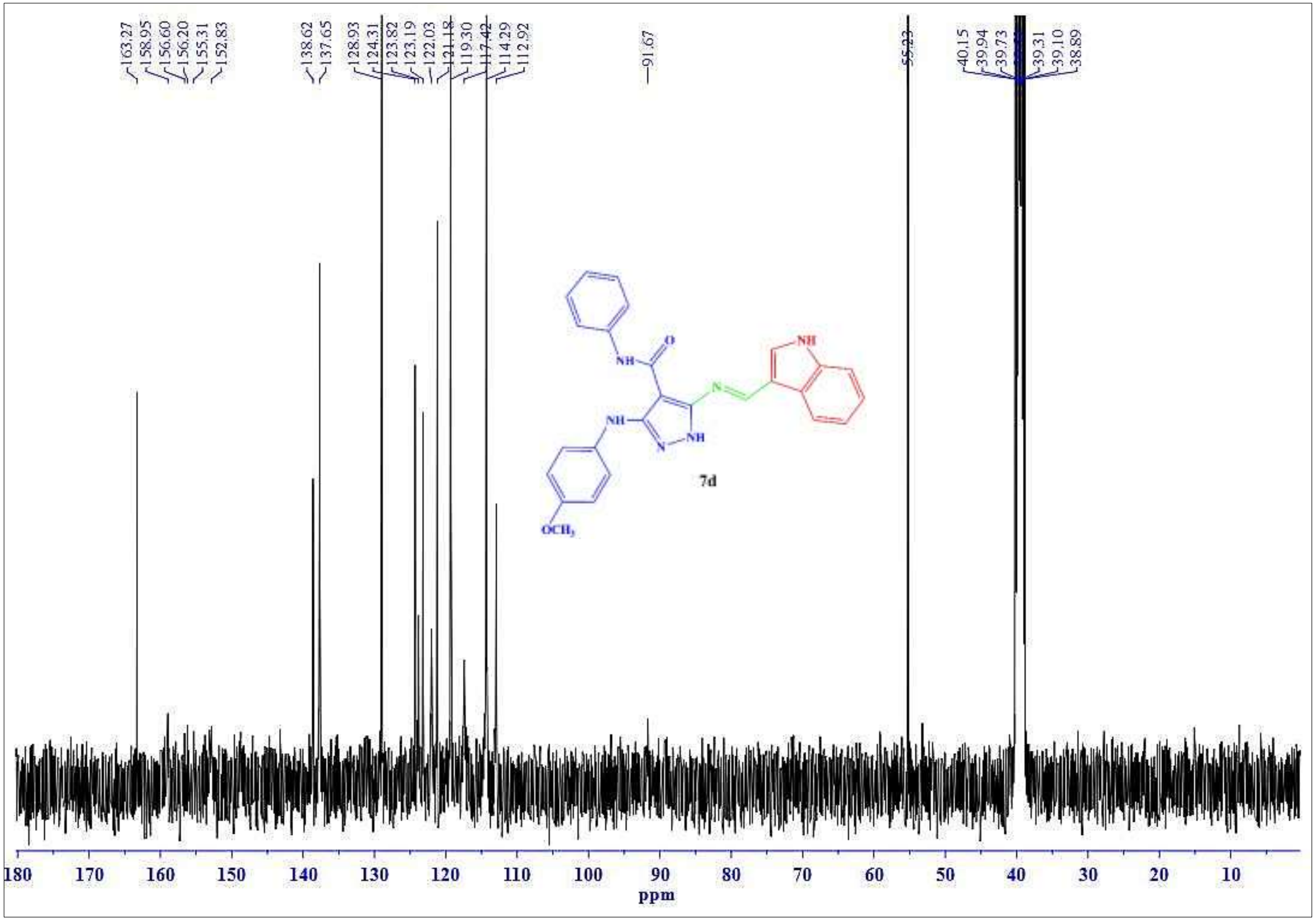

Figure S21: ^{13}C NMR Spectrum of compound **7d**

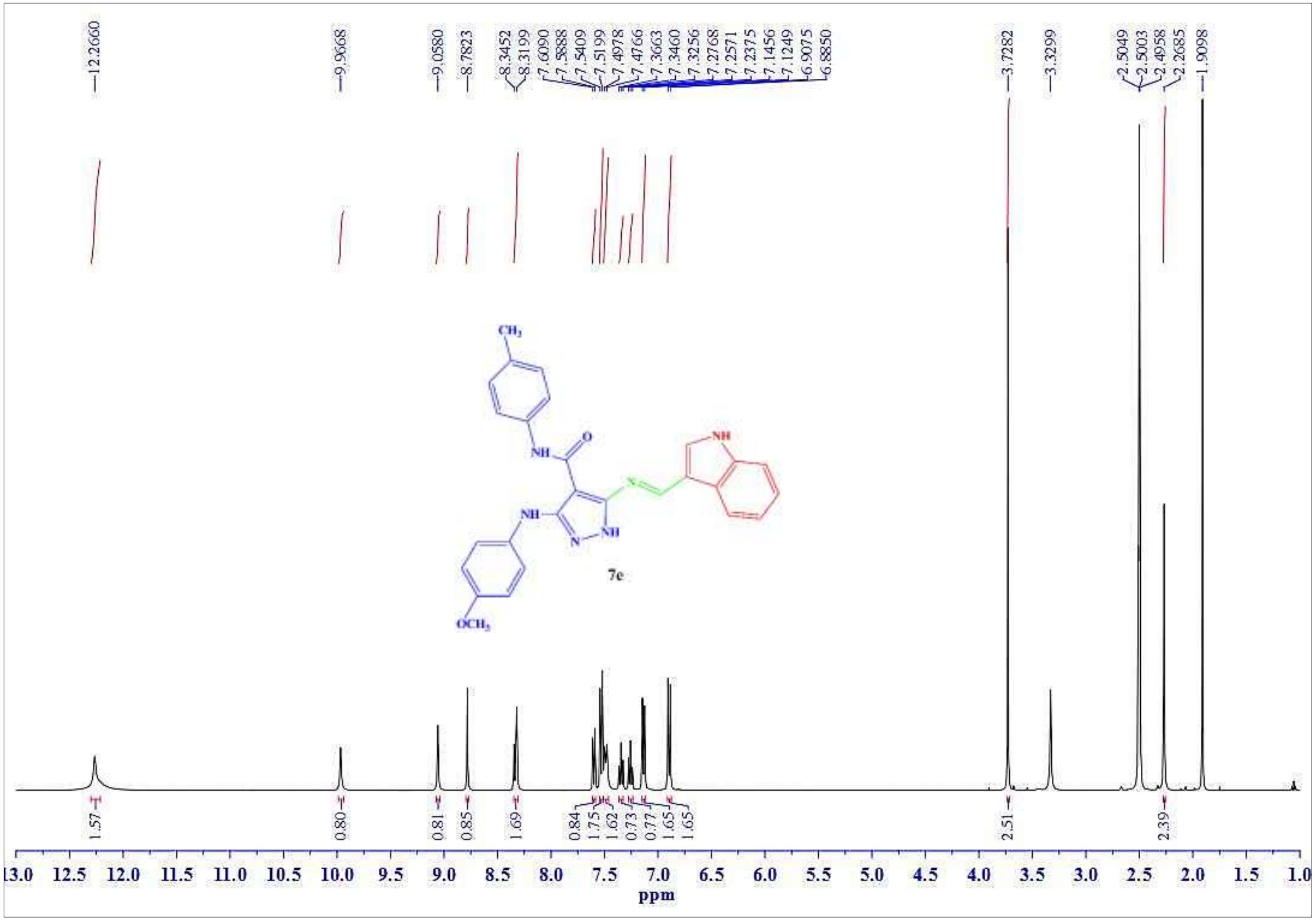

Figure S22: ^{1}H NMR Spectrum of compound **7e**

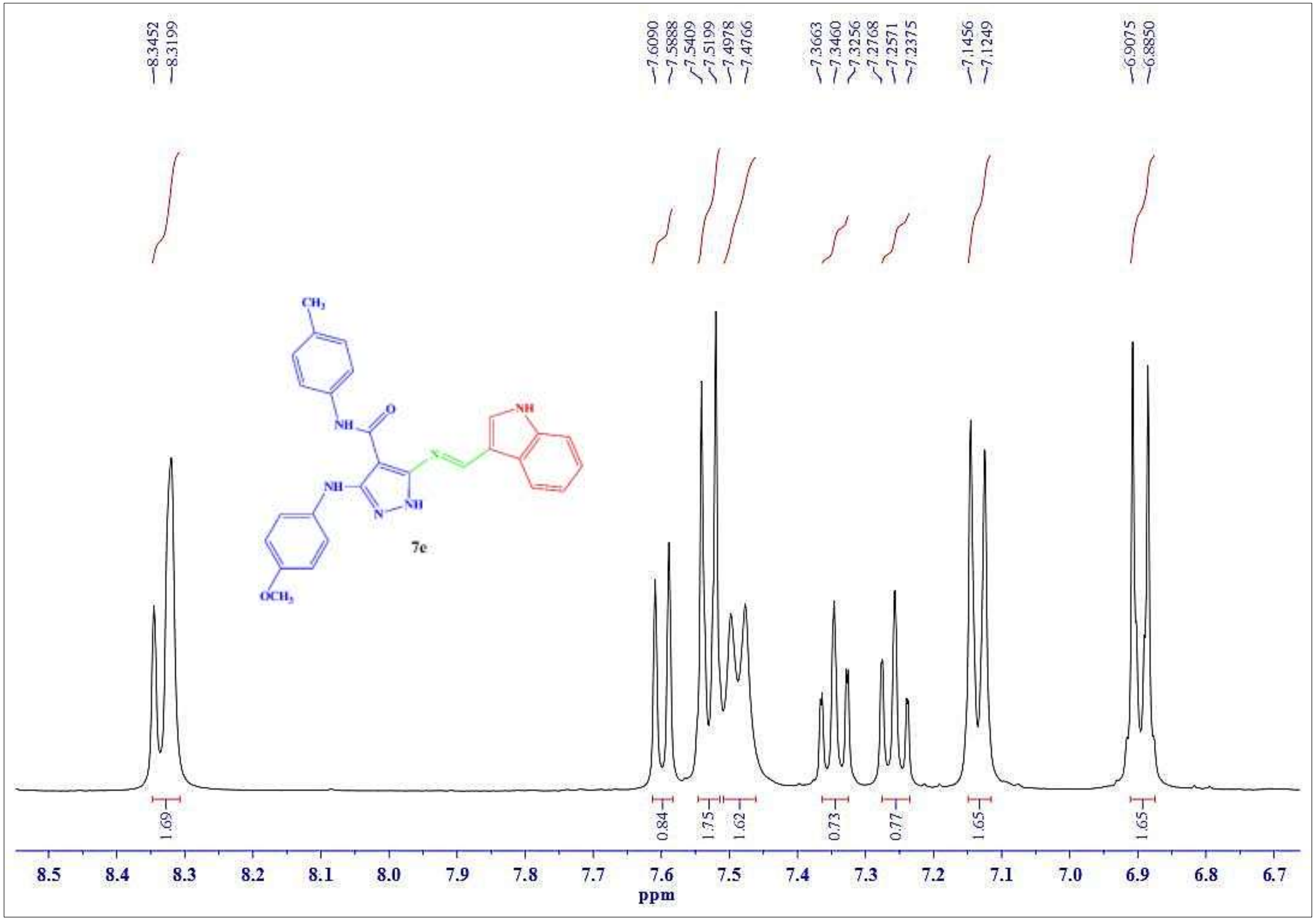

Figure S23: ^{1}H NMR Aromatic region spectrum of compound **7e**

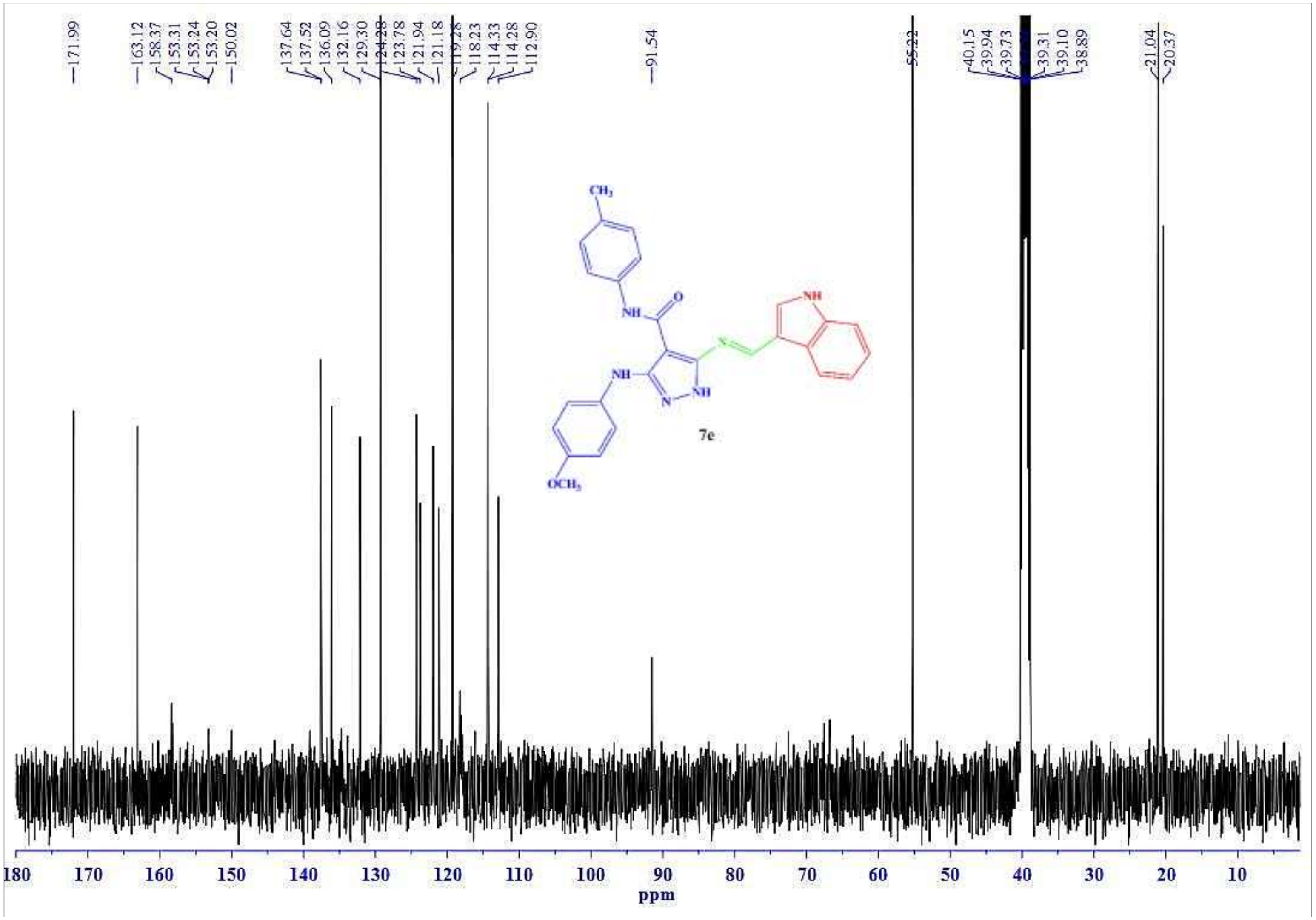

Figure S24: ^{13}C NMR Spectrum of compound **7e**
